# Budding Yeast Kinetochore Proteins, Chl4 and Ctf19, Are Required to Maintain SPB-Centromere Proximity during G1 and Late Anaphase

**DOI:** 10.1371/journal.pone.0101294

**Published:** 2014-07-08

**Authors:** Soumitra Sau, Sabyasachi Sutradhar, Raja Paul, Pratima Sinha

**Affiliations:** 1 Department of Biochemistry, Bose Institute, Kolkata, India; 2 Department of Solid State Physics, Indian Association for the Cultivation of Science, Kolkata, India; Florida State University, United States of America

## Abstract

In the budding yeast, centromeres stay clustered near the spindle pole bodies (SPBs) through most of the cell cycle. This SPB-centromere proximity requires microtubules and functional kinetochores, which are protein complexes formed on the centromeres and capable of binding microtubules. The clustering is suggested by earlier studies to depend also on protein-protein interactions between SPB and kinetochore components. Previously it has been shown that the absence of non-essential kinetochore proteins of the Ctf19 complex weakens kinetochore-microtubule interaction, but whether this compromised interaction affects centromere/kinetochore positioning inside the nucleus is unknown. We found that in G1 and in late anaphase, SPB-centromere proximity was disturbed in mutant cells lacking Ctf19 complex members,Chl4p and/or Ctf19p, whose centromeres lay further away from their SPBs than those of the wild-type cells. We unequivocally show that the SPB-centromere proximity and distances are not dependent on physical interactions between SPB and kinetochore components, but involve microtubule-dependent forces only. Further insight on the positional difference between wild-type and mutant kinetochores was gained by generating computational models governed by (1) independently regulated, but constant kinetochore microtubule (kMT) dynamics, (2) poleward tension on kinetochore and the antagonistic polar ejection force and (3) length and force dependent kMT dynamics. Numerical data obtained from the third model concurs with experimental results and suggests that the absence of Chl4p and/or Ctf19p increases the penetration depth of a growing kMT inside the kinetochore and increases the rescue frequency of a depolymerizing kMT. Both the processes result in increased distance between SPB and centromere.

## Introduction

The kinetochore (KT) is a multiprotein structure formed on centromere (*CEN*) DNA that links chromosomes and microtubules (MTs). It plays a crucial role in faithful segregation of replicated copies of a chromosome. The budding yeast KT contains about 60 different proteins which are organized into three (inner, central and outer) layers [1], [2], [3], [4]. The inner layer contacts the centromere while the outer layer connects the KT with the MTs. The central layer links the inner and outer layers. The Ctf19 complex of proteins lies in the central layer and contains three different subcomplexes identified by two-hybrid, coimmunoprecipitation and biophysical studies. These subcomplexes are COMA, consisting of Ctf19, Mcm21, Okp1 and Ame1 proteins [5]; Ctf3, containing Mcm22, Mcm16 and Ctf3 proteins [6]; the Chl4p-Iml3p subcomplex [7], [8]. In addition, Nkp1 and Nkp2 are also present in the Ctf19 complex [9]. In vertebrates, CENP-H, CENP-I, CENP-O, CENP-P, CENP-M and CENP-N are the members of constitutive centromere-associated network (CCAN) and considered as homologues of Mcm16, Ctf3, Mcm21, Ctf19, Iml3 and Chl4, respectively [3], [10]. Member complexes of the central layer are required by the spindle MTs for efficient initial capturing of the KTs to facilitate their transport to the spindle pole [11].

Studies have shown that apart from a brief interval of a few minutes in S-phase during their replication, centromeres stay attached to the MTs and cluster close to the spindle pole bodies (SPBs) throughout the cell cycle [12]. SPB and centromere distances are constrained in most of cell cycle [12], [13], [14], [15], [16], [17], [18], [19]. Using GFP-tagged *CEN4* and Spc42p, a spindle pole body protein, Dorn et al. have determined the average distances between SPB and centromere in the G1 phase as varying from 480 to 630 nm, with temperature varying from 25 to 37°C [18]. Using different centromeres it has been found that in late anaphase to telophase, centromeres are confined within 300 nm to less than 1000 nm from their SPBs [15], [16]. Loss of the KT function and addition of the MT poison nocodazole disrupt this proximity [20]. The biological significance of KT clustering is suggested to prevent chromosomes from entering the plane of cytokinesis and thus prevent unequal segregation of chromosomes [13]. A recent work by Richmond et al. implicates Slk19p in helping KTs to remain glued to each other in the absence of MTs which is suggested to assist the process of chromosome segregation upon recovery from MT disruption [21].

Stability of a dicentric plasmid in a KT mutant indicates weakened KT-MT interactions [22]. We have shown earlier that KT-MT interactions are compromised in mutants having defects in non-essential proteins of the Ctf19 complex [7], [23], [24], [25]. Since a kinetochore microtubule (kMT) exerts pulling and pushing forces on a KT towards or away from the SPB, it was expected that weakened KT-MT interactions would affect the centromere positioning in the nucleus. Since the KT protein complex is formed on a centromere, for the sake of simplicity we have used centromere and KT interchangeably while referring to distances from SPB. In this work we have determined that the proximity of centromeres to SPBs is indeed altered in mutant cells deficient in Ctf19 complex proteins. We measured distances between SPB and centromeres in late anaphase cells of the wild-type and mutant strains lacking Chl4 and/or Ctf19 proteins and found that centromeres of mutant cells lie, on an average, at greater distances from their SPBs than those of wild-type cells. Similar results were obtained for cells in G1 phase in that centromeres of mutant cells were scattered at distances from their SPBs which were longer than those of wild-type cells. Using a strategy to completely disrupt MTs, we show that the SPB-centromere proximity and distances are not dependent on protein-protein interactions between SPB and KT components. Furthermore, quantitative studies of the KT positioning based on the (1) independently regulated kMT dynamics, (2) polar ejection force on the chromosome balancing the kMT mediated poleward tension on the KT, and (3) altered kMT kinetics regulated by different loads represented by mutant and wild-type KTs has been carried out. The first of these rely on the freely adjusted kMT dynamics in the mutant compared to the wild-type and the third on the length and force dependent regulation of kMT dynamics. The KT positions predicted by the third model concur with experimental results.

## Materials and Methods

### Media and chemicals

All media, chemicals and enzymes are described in [7], [24], [26]. 3-aminotriazole (3-AT), nocodazole, alpha-factor, 4′, 6-diamidino-2-phenylindole (DAPI), phenylmethylsulfonylfluoride (PMSF), protease cocktail inhibitor, pepstatin A, leupeptin, aprotinin were from Sigma. Iso-propyl-1-thio-*β*-D-galactopyranoside (IPTG) and glutathione-Sepharose 4B beads were from Amersham Biosciences.

### Antibodies

Rat monoclonal (3F10) antibody directed against the HA epitope was from Roche Applied Science. Mouse monoclonal anti-GST (B-14) and mouse monoclonal anti-Myc (9E10) antibodies were from Santa Cruz Biotechnology, Inc. Rat monoclonal antibody directed against α-tubulin (YOL1/34) was from Serotec Ltd. UK. Alkaline phosphatase (AP)-conjugated goat anti-mouse immunoglobulin G (IgG), AP-conjugated rabbit anti-rat IgG and TRITC-conjugated rabbit (or goat) anti-rat IgG secondary antibodies were all from Sigma.

### Yeast strains and plasmids constructs

The strains used were AP22 (*MATα leu2-3,112 his3-11,15 ura3-52 trp1*) [27], PJ69-4A (*MAT* **a** *trp1-901 leu2-3,112 ura3-52 his3-200 gal4*Δ *gal80*Δ *LYS2::GAL1-HIS3 GAL2-ADE2 met2::GAL7-lacZ*) [28] and US3329 [*MAT* **a** *leu2::LEU2::tetR-GFP tetOX224::HIS3* (inserted 1.5 kb to the left of *CEN5*) *ura3 trp1 leu2 his3 ade2*] [29]. In this strain *CEN5* gets tagged with GFP and appears as a green dot under fluorescence microscopy. For the sake of simplicity we refer to this as *CEN5-GFP* [25]. All deletions/disruptions of *MCM16, CHL4, IML3, MCM21* and *MCM22* in these strains were carried out using appropriate deletion plasmids described before in [7], [23], [24], [30] or in supplementary methods (Text S1 in File S1). The deleted strains, their genotypes and references to deletion strategies are described in Table S1 in File S1. Plasmid constructions for two-hybrid studies, tagging of proteins with Myc, HA or GST epitopes, two hybrid study and GST pull-down assay are described in supplementary methods (Text S1 in File S1).

### Cell synchronization in G1 and G2/M phase of cell-cycle

The procedure for G1 arrest and subsequent release of cells in S-phase was carried out according to references [25], [31]. To arrest cells in G2/M, exponentially growing yeast cultures were treated with 15 µg/ml nocodazole (in 1% DMSO) and incubated with the drug for 90 to 120 minutes at 30^ο^C. This treatment resulted in the arrest of over 85% of cells in the culture.

### Cytology and microscopy

**Figure 1:**
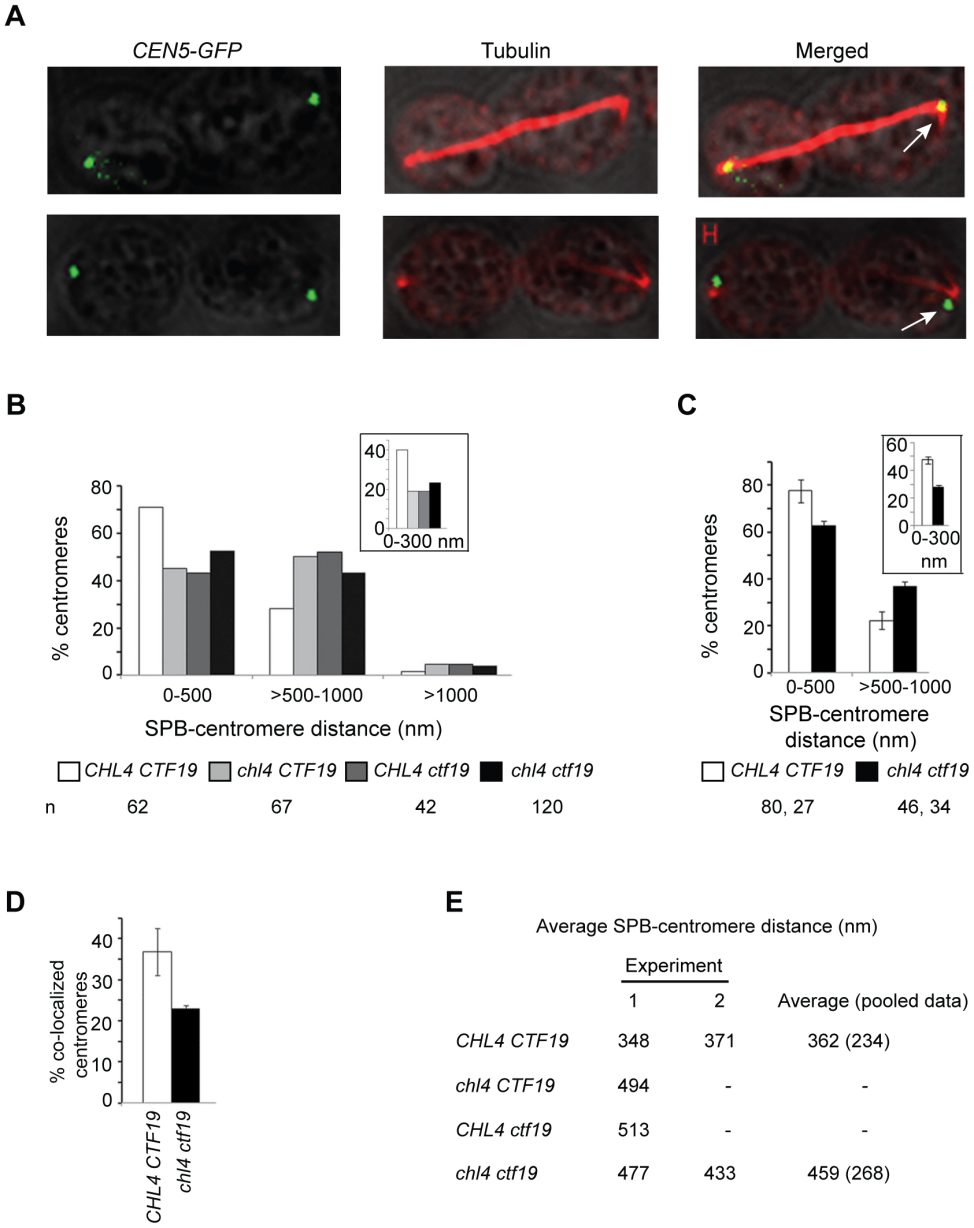
Average SPB-centromere distances are greater in mutant than in wild-type cells in late anaphase. US3329 (*CHL4 CTF19*; wild-type), US3329Δchl4 (*chl4*), US3329Δctf19 (*ctf19*) and US3329Δctf19Dchl4 (*chl4 ctf19*) cells, each carrying *CEN5*-*GFP* tag, were grown to log phase in YEPD medium at 30°C and then arrested at G2/M using nocodazole (15 µg/ml) for 90 minutes. Cells were fixed with formaldehyde after 30 minutes of release from G2/M, to maximize the population of anaphase cells. MTs and spindle pole bodies (SPB) were stained with anti-α-tubulin antibody, YOL1/34. Images were captured and analyzed by confocal microscopy. (A) Large budded cells having anaphase B spindles showing colocalization (upper panel) and non-colocalization (lower panel) of *CEN5* (green dot) with SPB (bright red spot at the end of the spindle, from where astral and nuclear MTs emanated). Arrows in merged panels indicate *CEN5*-SPB colocalization (upper panel) and non colocalization (lower panel). Scale bar, 0.5 µm. (B and C) 3D distances from the center of *CEN5*-*GFP* dot to the center of spindle pole were measured in cells having long anaphase spindles. Images were taken at 0.5 µm z-sections for (B) and at 0.2 µm z-sections for (C). For (C), two independent experiments were performed with wild-type and *chl4 ctf19* strains and the data represents averages obtained from these experiments, error bars indicating deviations from the average. For both B and C, ‘n’ refers to the number of centromeres analyzed and insets show % centromeres within 0–300 nm from their SPBs. (D) Histogram depicts percentages of colocalized *CEN5* and spindle pole obtained from (C). The bars are the deviations from mean values. (E) The average distances between centromere and SPB in wild-type and mutant cells, obtained from B (Experiment 1) and C (Experiment 2; pooled from two experiments) are summarized. Next column displays the average of Experiments 1 and 2. Standard deviations from average values are in parenthesis. ‘-‘ indicates not done.

**Figure 2:**
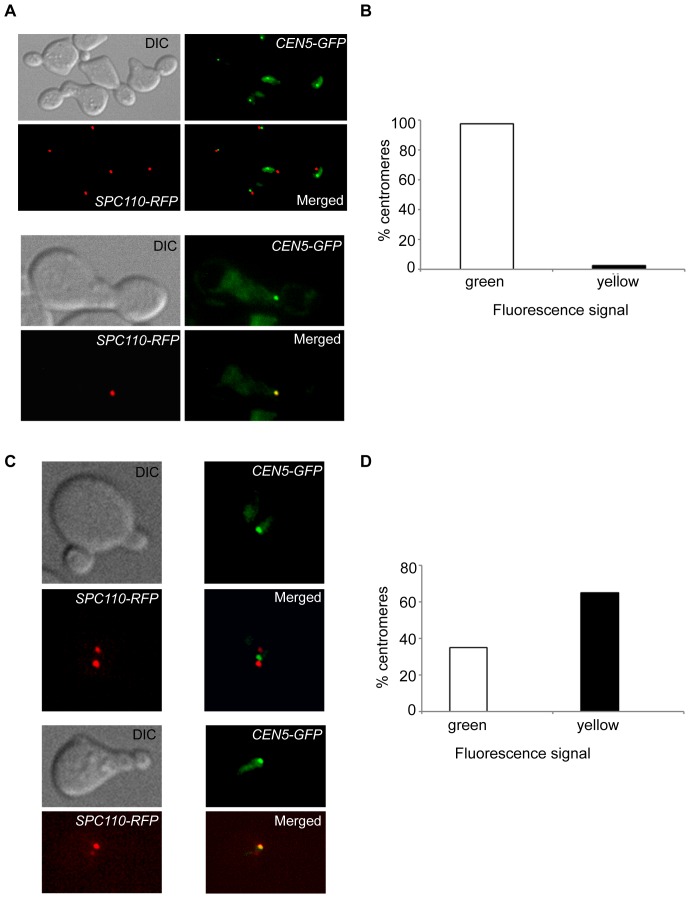
Protein-protein interactions between SPB and KT components do not contribute to SPB-centromere proximity. Exponentially growing cells of the wild-type strain PS1 (*CHL4 CTF19*), carrying its *CEN5* tagged with GFP and its Spc110p (an SPB protein) tagged with the RFP, were arrested in G1 by α-factor for 120 minutes. Thereafter, the cells were released from G1 arrest at 30°C in YEPD carrying nocodazole (15 µg/ml) or no nocodazole (control cells). Cells subjected to nocodazole were fixed with formaldehyde and processed for fluorescence microscopy 2 hours after release from G1 arrest. Control cells were fixed and processed similarly after 40 minutes of release from G1 arrest. (A) Upper two panels show a field of nocodazole-treated cells, each having its *CEN5* (green dot) non-colocalized with its SPB (red spot due to Spc110p-RFP). Lower two panels show the lone cell (one out of a total of forty analyzed) having its *CEN5* colocalized with SPB. (B) The bar diagram depicts percentages of centromeres which overlapped (yellow dots) and did not overlap (green dots) with SPB in the presence of nocodazole. (C) Fluorescence microscopy images of control (untreated with nocodazole) cells having non-overlapping (upper two panels) and overlapping (lower two panels) signals of *CEN5*-*GFP* and Spc110p-RFP (SPB). Note that the two SPBs (each tagged with Spc110-RFP) get separated from each other by MTs (not stained in this experiment) due to the absence of nocodazole. (D) The bar diagram represents percentages of centromeres showing overlapping (yellow) and non-overlapping (green and red) *CEN5* and SPB signals in S-phase in the presence of MTs. A total of 68 cells with two SPBs (indicated by two Spc110-RFP spots) were analyzed.

**Figure 3:**
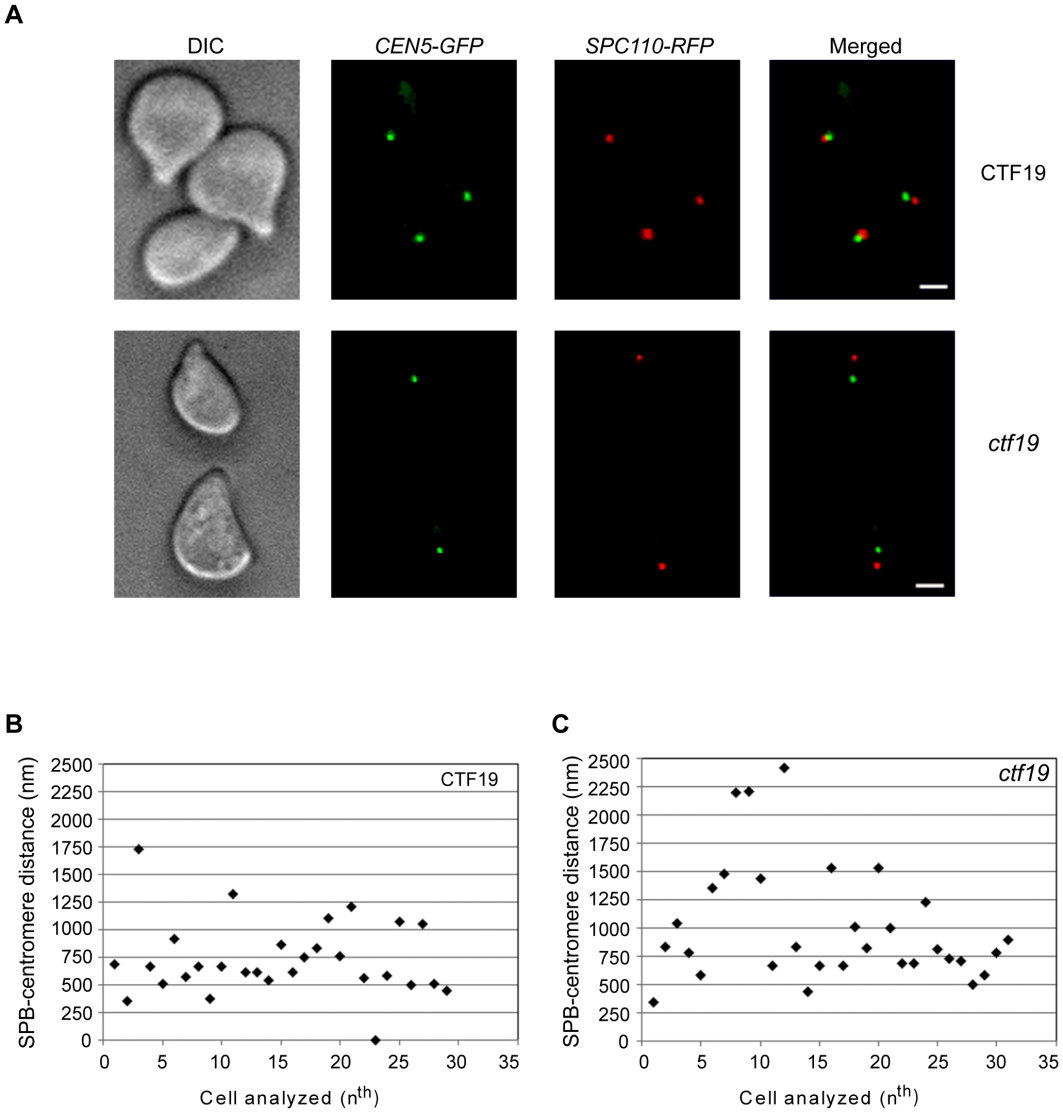
*ctf19* cells show increased average SPB-centromere distance at G1. PS1 (wild-type) and PS1Δctf19 (*ctf19*) cells were arrested at G1 using α-factor (10 µg/ml) for 120 minutes and fixed with 4% paraformaldehyde (PFA) for 10 minutes at RT. At least 20 z-sections of 0.25 µm were imaged. (A) G1 cells from PS1 (upper panel) and PS1Δctf19 (lower panel) are shown. Yellow or partial yellow indicates overlapping of SPB (Spc110p-RFP) and centromere (*CEN5*-*GFP*). Scale bars, 2 µm. (B) The 3D distance between SPB and *CEN5* was measured in G1 arrested cells and plotted. 29 cells of the wild-type and 30 cells of the mutant were analyzed.

For immunostaining (Figure 1 and Figure S3 in File S1), formaldehyde was added to a final concentration of 3.7% to the culture, for cell fixation, at room temperature (RT) for one hour. Thereafter, the cells were processed according to the published method [32]. Rat monoclonal α-tubulin antibody was used at a dilution of 1∶250 to stain MTs and spindle pole bodies. TRITC-conjugated rabbit or goat anti-rat IgG (1∶40 dilution) was used as the secondary antibody. The mounting solution was 3 µl of PD-DAPI solution (0.5 µg/ml DAPI in 90% glycerol containing 1 mg/ml of the anti-fade dye *p*-phenylenediamine). Images of immunostained cells were captured in 0.2 µm and 0.5 µm z-sections with a pixel spacing of 90 nm using a laser scanning confocal microscope LSM 510 Meta from Zeiss (Germany) which was equipped with an inbuilt software laser scanning microscope LSM 510 version 4.0 SPI. The objective used was plan-apochromat 100X/1.4 (NA) Oil DIC. To fix PS1 (*CEN5-GFPSPC110-RFP*) and its derivative mutant strain, cells were incubated with 4% paraformaldehyde (PFA) for 10 minutes at RT, washed once with 0.1 M KPO_4_/Sorbitol buffer(pH 7.5) and resuspended in the same buffer. Cells were imaged at 0.5 µm (Figure 2) and 0.% µm (Figure 3, Figures S4 and S5 in File S1) z-sections with a pixel-pixel of 129 nm using an Olympus BX-60 microscope at room temperature at 100X/1.3 (NA) Oil DIC, equipped with Photometrix Quantix camera (Roper Scientific) and MetaMorph 7.5 software (Universal Imaging Corporation). Upright fluorescence microscope was used instead of confocal microscope for the visibility of RFP fluorophores. Although we are aware that in late anaphase the KT stretches towards SPB to about 40 nm from the *CEN* DNA [33], we have used *CEN* and KT interchangeably in the manuscript for simplicity.

### Image analysis

The 3D distances (Figure 1) between centromere (GFP) and spindle pole (TRITC) was obtained using LSM 510 Meta confocal microscope. The sections with highest GFP and TRITC intensity were first selected from the series of z-stack images. The distances were measured by using 3D distance measurement tool of the LSM 510 confocal Axiovision software from the center of the green dot to the center of the spindle pole, the latter identified as a bulging structure at each end of the spindle from where astral MTs emanated. For Figure 3 and Figures S4 and S5 in File S1, the 3D distance between centromere (GFP) and spindle pole (RFP) was obtained following Cui et al., after deconvolving the images using MetaMorph 2D deconvolution software (nearest neighbors algorithm) [34]. First the sections with highest GFP and RFP intensity were identified. The vertical distance ‘a’ between the centromere and spindle pole was obtained from z-sections of nucleus, whereas ‘b’ being their horizontal displacement was determined by 2D projecting of the highest intensity stacks and measuring the 2D distance from center of GFP spot to the center of RFP spot. The 3D distance (*d*) was derived using the equation 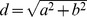.

## Results

### Centromere to SPB distance is increased in mutant cells

In the budding yeast, the centromeres stay clustered near the SPB almost throughout the cell cycle [20]. Disruption of the KT function and addition of the MT poison nocodazole disrupt this clustering but only partially [20]. Previous studies had reported reduced KT-MT interactions in the strains lacking non-essential proteins of the Ctf19 complex [7], [23], [24], [25]. We suspected that SPB-centromere distances might get affected in *chl4* and *ctf19* strains as absence of these proteins debilitates KT-MT interactions. For this, we determined the distances in late anaphase cells of the mutant and wild-type strains, when the KTs stay close to the SPBs.

For the experiment a wild-type strain (US3329) was used where *CEN5* was marked with GFP (see Materials and methods). Thus, each centromere appeared as a green dot when observed under a fluorescence microscope and its position was taken to specify the KT position in the cell. The distance between this GFP spot and the spindle pole body, stained with anti-tubulin antibody, was measured to compare the proximity of *CEN5* to SPB in late anaphase cells of US3329 and in each of the isogenic mutant strains *chl4*, *ctf19* and *chl4 ctf19*, constructed by deleting or disrupting *CTF19* and *CHL4* genes in US3329 (Table S1 in File S1). Log phase cells growing in YEPD medium at 30°C were arrested in G2/M as described in Materials and methods. The cells were synchronized in G2/M so that upon subsequent release from arrest, the population of cells in anaphase could be maximized. The cells were released from arrest in YEPD for 30 minutes (to maximize the percentage of late anaphase cells), fixed with formaldehyde and the MTs and SPBs were stained with anti-tubulin antibody as described in methods. The stained cells were observed under a confocal microscope for GFP dots and mitotic spindles. In the first experiment, images of GFP dots and the spindle poles were taken in 0.5 µm z-sections. The 3D distances (see Materials and methods) of *CEN5* (GFP dots) from the spindle poles were measured both in the wild-type and mutant cells having long, late anaphase spindles (Figure 1, A and B), the average length of the spindles being 8.1 µm. Figure 1B shows that while 75% of the wild-type centromeres were contained within 500 nm and almost all of the remaining 25% were within 1000 nm, mutants had centromeres which were, on an average, further away from the SPB. About 50% centromeres of the mutant strain were at distances greater than 500 nm but most were again contained within 1000 nm. There was no additive decline in centromere-SPB proximity in the double mutant, as compared to the single mutants. Within this window of 500 nm, about 40% of wild-type centromeres were within 300 nm from the SPB as compared with about 20% of the mutant strains (Figure 1B, inset). To further confirm the results obtained with 0.5 µm z-sections, two independent experiments were done subsequently with the wild-type and *chl4 ctf19* cells, imaged at 0.2 µm intervals. The results obtained from these two sets (Figure 1C) were very similar to those from Figure 1B in that a higher fraction (∼80%) of wild-type centromeres were within 500 nm of SPB as compared to ∼60% centromeres of the mutant strain. The proximity of the centromeres to SPBs in mutant and wild-type strains was also determined by counting the fraction of centromeres that displayed overlaps with the spindle pole bodies in 0.2 µm sections. A complete or partial overlap of SPB (red) with *CEN5* (green) signals was visible as yellow or yellow-green signals (Figure 1A) in 40% of wild-type and 25% centromeres of the mutant strain (Figure 1D). Over 95%of these centromeres were contained within a distance of 300 nm from SPBs and the remaining were all within 350 nm, which compares reasonably well with the actually observed values for the percentages of wild-type and mutant KTs (47 and 27 respectively) lying within 300 nm (Figure 1C, inset). The average SPB-centromere distances in the wild-type and mutant strains were calculated and it was found that the distances in the mutant strains were increased by 20 to 30% over the wild-type distances (Figure 1E). The average SPB-centromere distances of the pooled results were 362 nm for the wild-type and 459 nm for the mutant strain. These observations convincingly show that centromeres of the mutant strain are clustered further away from the spindle poles than the wild-type centromeres.

### Protein-protein interactions between components of SPB and KTs do not contribute to maintain SPB-KT distances

Using two-hybrid interactions and GST-pull down assays we have shown that both Chl4 and Ctf19 proteins physically interact with a spindle pole body protein Bbp1p (Texts S2 and S3; Figures S1 and S2 in File S1). Several other proteins from SPB also show physical interactions with KT proteins (Table S2 in File S1). These interactions could possibly help to maintain KT-SPB distances in wild-type strains and contribute to chromosome segregation by facilitating the process by which KTs are brought close to the SPB [13], [16], [35], [36]. Therefore, a disruption of interactions between Chl4 and Ctf19 proteins with Bbp1p could conceivably have led to increased distances between SPB and *CEN5* as observed above. On the other hand, both *chl4 and ctf19* are documented to cause weakened KT-MT interactions [25], [35], [37]. A reduction in poleward forces due to weak KT-MT interactions could also increase SPB-KT distances in mutant cells. It was reasoned that if KT-MT interactions were mainly responsible for the maintenance of KT-SPB distances in cells, in the absence of MTs, wild-type KTs would not be any closer to SPBs than mutant KTs. When MTs are depolymerized by nocodazole, SPBs can duplicate but not separate and most are visible as single spots under fluorescence microscopy [38]. To determine SPB-KT distances in the wild-type and mutant strains in the absence of MTs, exponentially growing cells were treated with nocodazole for two hours and stained for α-tubulin. At this time point about 30-40% cells showed tubulin staining of which about 70% displayed a single fluorescent spot of SPB. Cells with single spots, (SPBs having no visible MT-like fuzzy projections) and single green dots (representative of cohesed sister chromatids) were analyzed for the separation between SPB and the KTs (Figure S3A in File S1). The fractions of cells having SPB-KT distance as ≤300 nm were found to be 22% and 12% respectively for the wild-type and mutant cells (Figure S3B in File S1). Thus, wild-type KTs were observed to be closer to SPBs than mutant KTs in the absence of MTs. This result indicated that factors other than MT-KT interactions were playing roles in promoting KT-SPB proximity in the wild-type. However, short MTs (about 100 nm length) are relatively more resistant to depolymerization by nocodazole [38]. Therefore, it could be argued that a fraction of KTs persisting within 100 nm from SPB after nocodazole treatment were bound to short MTs resistant to nocodazole treatment. Since normally a greater fraction of wild-type KTs lies within 300 nm of SPB [Figure 1, B and C (insets)], it is possible that a relatively greater fraction had remained attached to MTs after nocodazole treatment. Therefore, there was a need to detach KTs from short MTs completely before concluding about the roles of protein-protein interactions in maintaining SPB- KT distances.

The only phase of the cell cycle where KTs detach themselves from MTs to distances of about 1000 nm is a small window in the early S-phase just prior to centromere replication [12]. After this, the KT gets captured again by a MT and reaches the vicinity of SPB by the action of both Kar3p (lateral pulling) and Dam1 complex (end-on pulling) [12]. To see if intact wild-type KTs could cluster at SPBs in the absence of MTs, cells synchronized in G1 phase of the cell cycle were treated with nocodazole for fifteen minutes and then released from G1 arrest in nocodazole-containing medium. The cells were kept shaking in this medium for two hours. To reach the SPBs in the absence of MTs, the detached KTs would have to diffuse passively to the SPBs or rely on some active mechanism involving protein-protein interactions between KT and SPB components for clustering. The nocodazole-treated cells were stained with anti-α-tubulin antibody and scored for overlaps (partial or full) between the GFP dots and the spindle pole bodies, indicative of distances less than 300 nm. However, in most cells the SPBs did not stain at all or the staining was very poor (data not shown). It is likely that nocodazole can more efficiently disrupt short MTs which are not attached to KTs (as in this experiment) compared to those which are attached at the time of nocodazole treatment (previous experiment- Figure S3 in File S1). Therefore, another strain (PS1), which had its SPB protein Spc110 tagged with the red fluorescent protein (RFP), was used for the same experiment. G1-arrested cells of PS1 were treated with nocodazole as described above and the overlaps of GFP and RFP signals were analyzed. Interestingly, even after two hours in nocodazole, only one of 40 cells had an overlap of GFP-RFP fluorescence generating a yellow signal. In the remaining cells, only green and red dots were visible without overlaps (Figure 2, A and B). When the same cells were released in S-phase without nocodazole treatment, after 40 minutes of release 45% of small-budded cells had two SPBs indicative of entry into S-phase. Of these, over 65% had overlaps of GFP and RFP signals (Figure 2, C and D). Taken together, these results show that physical interactions between KT and SPB components do not help to establish SPB-*CEN* proximity. Therefore, the increased SPB-KT distances observed in *chl4*, *ctf19* and *chl4 ctf19* mutants were due to weakened KT-MT interactions in these strains.

### Mutant cells in the G1 phase exhibit longer SPB-centromere distances

It was of interest to see if the increase in SPB-centromere distances in the mutant cells was specific to anaphase but was also present in G1 cells. In anaphase, chromosomes are in uniform velocity in the nucleus, moving with the spindle poles towards the opposite ends of the dividing cell. During this motion they resist viscosity and residual cytoskeletal network of the spindle apparatus. In G1, these characteristics are absent. Therefore, net force experienced by the chromosome could be different in the two phases. Any differences in centromere positioning would show that a loss of non-essential components of the Ctf19 complex affects centromere forces in interphase as well. For this experiment the wild-type strain (PS1) and its mutant derivative *ctf19* (PS1Δctf19), obtained by deleting *CTF19* in PS1 (Table S1 in File S1), were arrested in the G1 phase using α-factor. We chose the single mutant because there were no significant additive or synergistic differences between the single and double mutants in centromere positioning in anaphase cells. Fixed cells were analyzed for distances between the GFP and RFP dots as described under the Materials and methods section.

The SPB-centromere distances in the G1 phase were again greater in the mutant *ctf19* than in the wild-type strain. The averages for the two strains were 730 nm (wild-type) and 1016 nm (*ctf19*). Although we obtained somewhat higher distances than 500–600 nm reported by Dorn et al., the fact remains that centromeres from the mutant strain were scattered further away from SPBs than those from the wild-type strain (Figure 3, A and B) [18]. Therefore, the *ctf19* deficiency alters forces on the KT such that the net pull on the KT towards SPB is decreased.

As stated before (Introduction), both *chl4* and *ctf19* mutations cause weakening of KT-MT interactions. This leads to the concern that nocodazole treatment could further exacerbate the defect in the KT that may, even after washing away of the drug, adversely influence the SPB-centromere distances. Similarly, it may also be argued that α-factor could modify MT characteristics [18]. To address these concerns we measured SPB-*CEN* distances in anaphase cells not treated with nocodazole and in exponentially growing G1 cells from the cultures of wild-type (PS1) and *ctf19* mutant (PS1Δctf19) strains. Cells, whose spindles were greater than 5 µm, as measured by the separation between their spindle poles, were taken as anaphase cells. The average spindle lengths for the wild-type and mutant cells were 7.24±1.05 and 7.58±1.38 µm respectively. G1 cells were identified as round unbudded cells. The results from these experiments showed the same trend as shown by nocodazole treated anaphase cells (Figure 1) and α-factor treated G1 cells (Figure 3). Thus, in untreated cells also, a noticeably larger proportion of wild-type kinetochores were found to lie within 400–500 nm of their SPBs, as compared to their mutant counterparts (Figures S4 A–B and S5 A–B in File S1).

### Force and Length dependent regulation of kMT dynamics explain differences in wild-type and mutant KT positions in anaphase and/or G1 cells

Having observed altered SPB-KT separation in the mutant, we sought to understand how this positioning relates to the absence of Chl4 and/or Ctf19, the non-essential KT proteins. The simplest model one can imagine for the mutant is that the non-essential KT proteins possibly affect at least one of the dynamic instability parameters of kMTs, e.g., perhaps the catastrophe frequency is slightly lowered or the rescue frequency is slightly raised in the mutant cell. This assumption does not impose any mechanical constraint on the kMT. The result is reported in the supplementary text (Text S4 in File S1). Assuming constant values for parameters of kMT dynamics during anaphase, first the probability distribution of KT-SPB distances is obtained for the wild-type (Figure S6 in File S1). Then, by carefully varying catastrophe and rescue frequencies, we obtain probability distribution function for the mutant as well. Both these distributions stretch well beyond the typical diameter of the yeast nucleus (∼2 µm). Then we looked into a second hypothesis (Figure S7 in File S1) that incorporates the “polar ejection force” [39], [40] as detailed in Text S5 in File S1. This force is primarily generated by the MTs, growing from the SPB and impinging upon the chromosome arm at random instants. The “polar ejection force” is counter balanced by the poleward tension between KT and SPB. Simulation data obtained with realistic parameters suggest that the position of the KT is not consistent with the experiment unless the simulation is manipulated with unrealistically large viscous drag coefficients (∼100 pN-s/µm) and with hundreds of nuclear MTs (Figure S7 in File S1). Therefore, these models do not seem to be appropriate for the current purpose.

It has been proposed by several research groups that KT positioning is dependent on the kMT dynamics [18], [41]; further, it is well established that kMT can generate sufficient force that can displace chromosomes [42], [43]. In general, depending upon the dynamical state, MT has the ability to both pull and push on a given object [42], [44], [45]. In other words, while polymerizing, kMT can generate a ‘thermal ratchet force’ away from the pole and while depolymerizing, it can produce poleward tension on the chromosome [42], [44], [45]. Thus, stochastic growth and shrinkage of kMT within the KT cylinder are incorporated in this framework to successfully predict the experimental results [46], [47]. In contrast to unconstrained MT dynamics (discussed in Text S4 in File S1), here we assume that KT positioning is rather regulated by force and length dependent kMT dynamics. Here we implement the concept that an external load and overall length modifies MT's dynamic instability parameters, viz catastrophe frequency (*f*_c_), rescue frequency (*f*_r_), growth velocity (*v*_g_) and shrink velocity (*v*_s_) [48], [49], [50]. The model has been simulated for various forms and magnitudes of dynamical parameters relevant to a kMT. These are discussed in the following model variants by regulating (A) the catastrophe and (B) the rescue frequencies of the kMT independently.

#### (A) Regulation of catastrophe frequency

**Figure 4:**
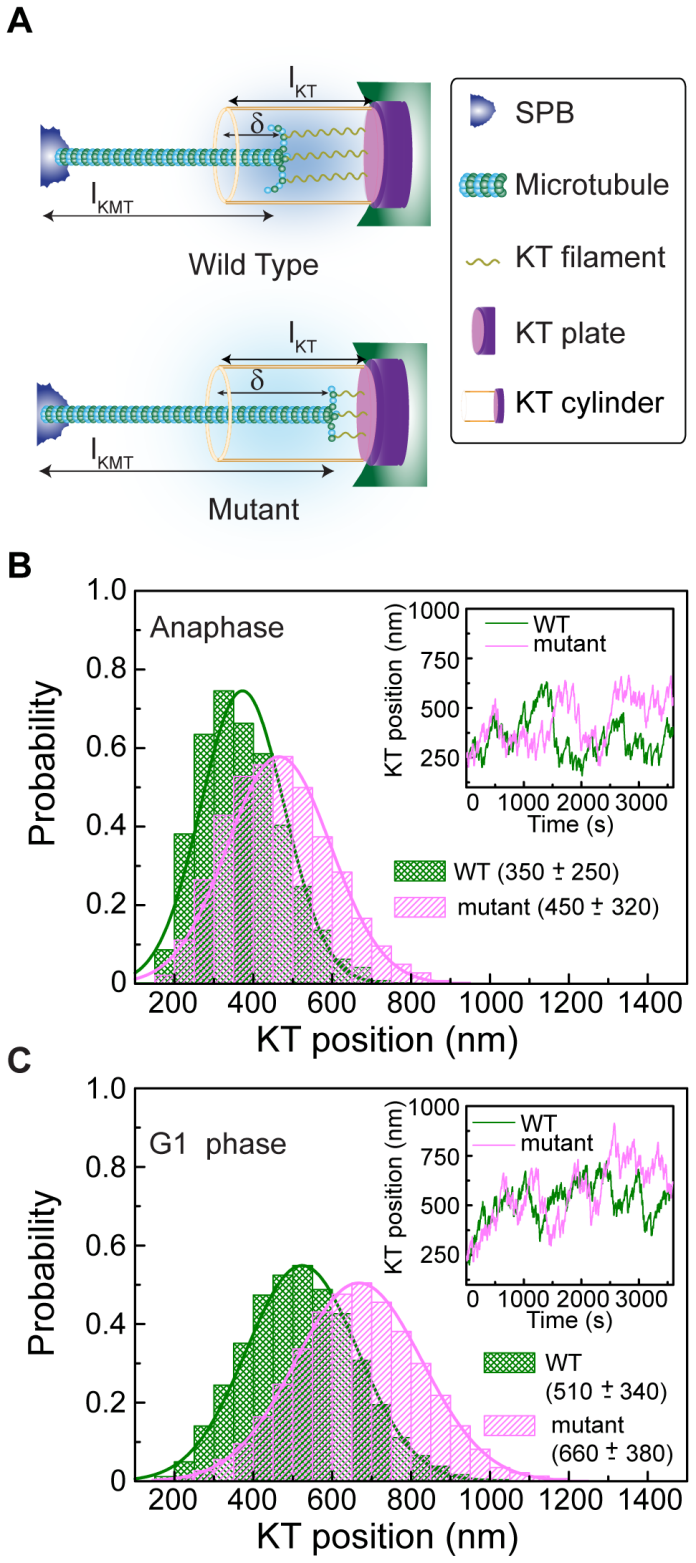
Altered catastrophe rate of kMT predicts KT positioning. (A) Schematic representation of kMT-KT interaction, showing various ingredients used in the model. In this model, the catastrophe events of the kMT depend on the applied resistance, instantaneous length (*l*_kMT_) and depth of penetration (*δ*). Mutation weakens the effective stiffness of the KT fibrils (represented by springs), thereby enhances the kMT polymerization. This effectively pushes the mutated KT away from the SPB. (B) Probability distribution of the KT position measured from the SPB during anaphase. Green and magenta (color online) represent wild-type (WT) and mutant distributions, respectively. The average distances of the KT from the SPB are ∼350 nm for the wild-type and ∼450 nm for the mutant. Shifting of the mutant distribution to the right signifies a larger KT distance from the SPB. Inset shows the temporal change in the KT position for WT (green) and mutant (magenta). (C) Probability distribution of the KT position during G1 phase, plotted in the same manner as in (B). The average distances of the KT from the SPB are ∼510 nm and ∼660 nm for the wild-type and the mutant, respectively. Inset showing the time dependent position of the KT for both wild-type and mutant.

*In vitro* experiments suggest that diminishing tubulin assembly at the growing end of an MT due to the presence of a barrier increases the catastrophe rate [48]. In addition, it has also been shown that a longer MT undergoes catastrophe more often than a shorter one due to aging, possibly induced by molecular motors of the kinesin family [50], [51]. Therefore, the catastrophe frequency can be expressed as a function of the load force and kMT length in the following manner [49]:
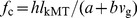
(1)where, *h* is a parameter adjusted to set the optimal length of MT beyond which it undergoes a spontaneous catastrophe. Considering *h*∼100.0/µm, a catastrophe length of MT *∼*1 µm can be achieved, which is the radius of yeast nucleus. *l*_kMT_ is the instantaneous length of the kMT, *a* is a constant (see Table 1) and b is calculated using: 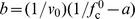. Here, *v*_0_ and *f*_c_^0^ are the unconstrained growth velocity and catastrophe frequency of the kMT, respectively. The model also assumes a constant rescue frequency *f*_r_^0^. Several *in vitro* experiments have suggested that a growing MT slows down at an exponential rate when suppressed by a load force *F* [48], [49], [52], leading to growth velocity:
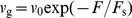
(2)The sensitivity force *F*_s_ ensures a rapid drop of the kMT growth velocity when the load exceeds this value. The model is schematically shown in Figure 4A. For the sake of simplicity, the KT is compared with a cylindrical object that grows outward from the centromeric region. The KT plate is the region of the inner cylinder attached with the centromere while the free end of the cylinder represents the outer KT. Majority of the load experienced by the growing kMT tip arises from the resistance most likely offered by various protein complexes and KT filament connecting the kMT tip with the KT plate [53]. KT fibrils are considered to act like elastic springs and mechanically resist the kMT polymerization [41]. The load force increases linearly as the kMT tip penetrates deeper into the KT cylinder, i.e., *F = kδ*, where *k* is the effective force constant of the KT fibrils and *δ* is the depth of penetration of the kMT tip inside the KT cylinder.

**Table 1:** Model Parameters.

Abbreviations	Meaning	Values used	Reference
*v*_0_	Unconstrained growth velocity	0.79 µm/min a	[54]
		0.85 µm/min b	
*v*_s_	Depolymerizing velocity	0.86 µm/min a	[54]
		1.04 µm/min b	
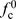	Catastrophe frequency	0.0109/s a	[54]
		0.0104/s b	
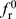	Rescue frequency	0.0089/s a	[54]
		0.0130/s b	
*l*_KT_	Length of the KT	0.1 µm	[67]
*A*	Stalled period of MT at *v*_g_ = 0	24 s	[48], [49]
*F*_s_	Sensitivity of MT growth force	1.7 pN	[49]
*K*	Force constant of KT fibrils(for WT/mutant)	55/40 pN/µm	[68]
*κ*	Force constant of kMT-KT coupling springs (for WT/mutant)	37.5/45 µm^−1^	[44]

a Values used for anaphase.

b Values used for G1.

The MT dynamical parameters obtained from Wolyniak et al. for G1 and preanaphase are used for simulation of G1 and anaphase [54]. We initiate the simulation with SPB at the origin and placing the KT at an arbitrary distance within 1 µm from the SPB. The probability of switching (*P*_switch_) between catastrophe and rescue events at each “time step” is derived using equation [41], [49], [55], 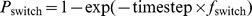, where *f*_switch_ corresponds to the catastrophe (*f_c_*) or the rescue (*f*_r_) frequency determined by the state of the MT. Although switching events between different dynamic states may occur in finite time scale, for simplicity we assume such events to occur instantaneously.

Since our model is developed to predict the KT position in anaphase and G1, MT-KT attachment is a preexisting condition in the model assumption. A detailed discussion of how this attachment can be established is discussed in the literature [44]. As the kMT penetrates inside the KT cylinder, it experiences a resistance offered by the KT fibrils. A growing kMT compresses the spring-like KT fibrils and transmits force into the KT. While in the polymerizing state, load on the kMT increases linearly; however, at the same time catastrophe frequency also increases exponentially (Equation 1 & 2), eventually leading to a catastrophe. Shrinking kMT is also capable of generating strong force [45]. kMT protofilaments splay off spontaneously during depolymerization (see Figure 4A) due to the release of strain energy stored in the curled protofilaments. Protofilaments mechanically anchor with the KT via spring-like coupler, possibly formed by Dam1/DASH ring complex [44]; thereby transmitting a poleward tension into the chromosome. The coupler ensures that the kMT tip remains within the KT cylinder by increasing the rescue frequency of the kMT [41], [56].

For the mutant cell, we assumed that due to the change in protein configuration within the KT cylinder, resistance on the kMT is reduced. Needless to say, the reduced load on the polymerizing kMT provides more freedom for the free tubulin monomers to attach to the plus end of the kMT. Our model incorporates this feature by decreasing the force-constant *k* of the KT fibrils. Thus, enhanced kMT polymerization within the mutant KT pushes the chromosome with a stronger force compared to the wild-type.

Transmission of force to the KT depends upon its interaction with the kMT. In other words, the actual force transmitted to the KT is a function of the efficiency of the KT-protein machinery that physically links kMT with the KT. We consider that the efficiency of this linking protein complex *γ* is less than unity (i.e. 0<*γ* <1) and simulate the model for various values of *γ* within this range [44]. Unless otherwise specified, all of our data presented here is obtained using a value of *γ* ∼0.4 [44], [57]. Unconstrained kMT dynamical parameters during anaphase and G1 are presented in Table 1 and in Table S3 in File S1. We find that the mean position of the KT and its fluctuation around the mean position increases with the efficiency (Figure S8 in File S1). Distance of KT from the SPB in anaphase is plotted against time in Figure 4B, showing results for both wild-type and mutant cells. We observe that the KT oscillates around the mean positions, which are ∼350±250 nm in the wild-type cell and ∼450±320 nm in the mutant cell, away from the SPB. These results are in reasonable agreement with our experimental data. Similar results for the G1 phase is plotted in Figure 4C. The SPB-KT distances ∼510±340 nm (wild-type) and ∼660±380 nm (mutant) also agree with the experimental data.

Equation 1 together with Equation 2 clearly implies that the catastrophe frequency of a kMT increases with its length and the load applied on it, indicating an enhanced polymerization and subsequent elongation of a weakly constrained kMT. Considering the hypothesis that a configurationally different KT protein complex in the *chl4* and/or *ctf19* strain relaxes the constraints on the growing kMT, polymerization activity of the kMT is increased. This essentially pushes the KT further apart, leading to an increased separation between KT and SPB in the *chl4* and/or *ctf19* mutants.

#### (B) Regulation of rescue frequency

**Figure 5:**
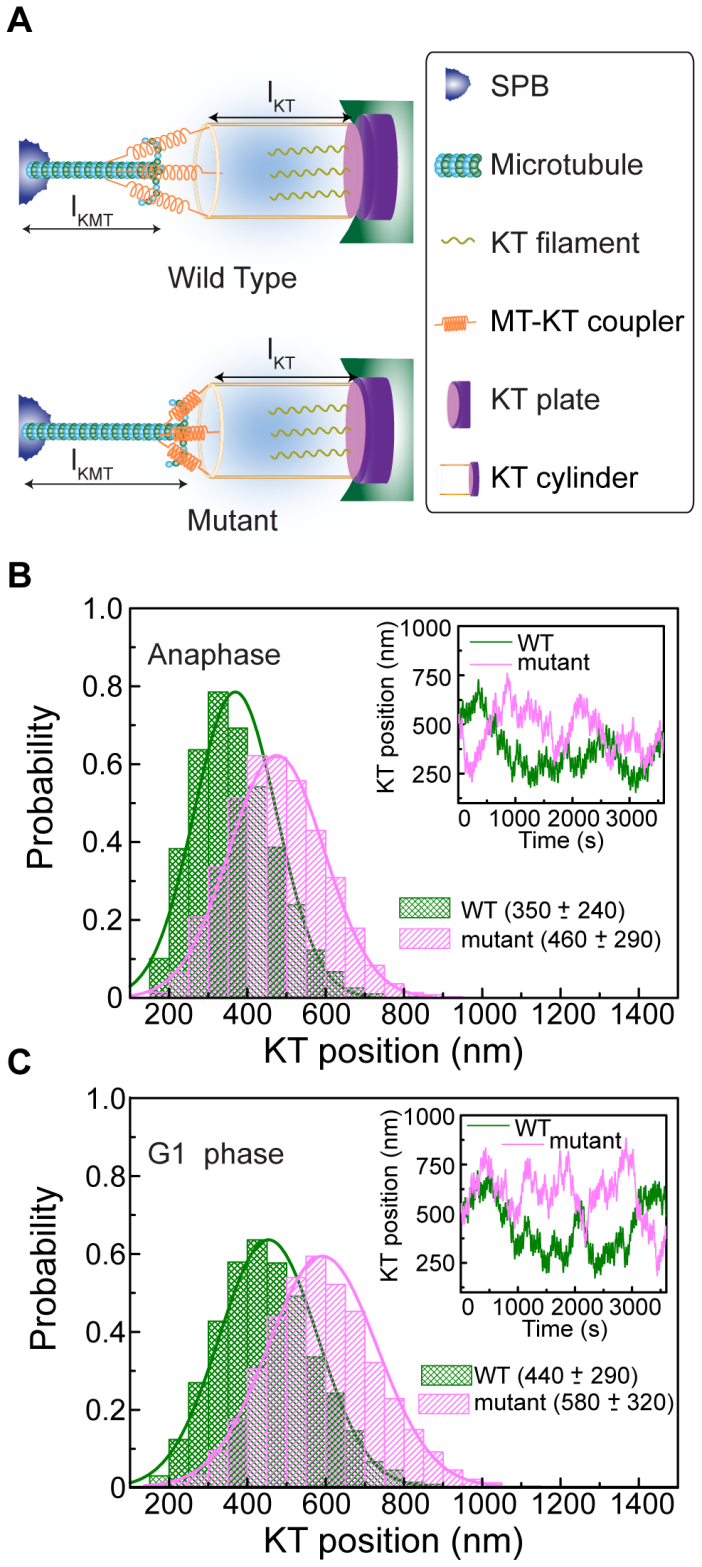
Altered rescue frequency of kMT predicts KT positioning. (A) Schematic is similar to Figure 4A. In this approach, the rescue frequency of the kMT is regulated by the tension applied on the kMT mediated by couplers. Mutation makes the couplers stiffer and thereby generates higher tension at the kMT tip. Thus in the mutant cell, frequent rescue of the kMT increases the equilibrium distance between SPB and KT. (B) Probability distribution of the KT position during anaphase, similar to Figure 4B. The average distances of the KT from the SPB are ∼350 nm for the wild-type and ∼460 nm for the mutant. Temporal change in the KT position for the WT (green) and the mutant (magenta) is shown in the inset. (C) Probability distribution of the KT position during G1 phase, plotted in the same manner as in (B). The average distances of the KT from the SPB are ∼440 nm and ∼580 nm for the wild-type and the mutant, respectively. Inset displays the time dependent position of the KT for both wild-type and mutant.

Contrary to the previous model, in which the catastrophe of a kMT is altered by the applied load and instantaneous length, the current scenario considers the rescue frequency to be tension dependent [41], [58]. A schematic description of the model is shown in Figure 5A. Here, the catastrophe frequency is assumed to be load independent and therefore Equation 1 is modified as 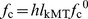 by removing the load dependent denominator. *h* is an adjustable parameter mentioned in the previous section and *l*_kMT_ is the length of the kMT. It is noteworthy that without the length dependent prefactor *hl*_kMT,_ an unconstrained MT on the average grows longer than a micrometer, which is well beyond the average distance between SPB and KT. The tension dependent rescue frequency (*f*_r_) [41], [55] is given by 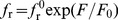
(3)where, 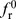 is the unloaded rescue frequency and *F* is the tension applied on the kMT tip. The applied tension is normalized by a characteristic force *F*_0_ that increases the rescue frequency e-fold. In this model, we consider a strong attachment between the kMT tip and the KT cylinder by protein complexes (henceforth coupler) that resist complete detachment of kMT [41], [44]. We model the couplers as linear spring-like objects that apply tension on a depolymerizing kMT and strongly resist its escape from the KT cylinder. As the kMT depolymerizes, the tension on the tip builds up linearly due to the stretching of the couplers as per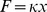, where *κ* is the force constant and *x* is the amount of stretching. This leads to an exponential increase in the rescue frequency *f*_r_ in Equation 3. Note that, for the sake of a dimensionless argument of the exponential in Equation 3, *κ* is normalized by *F*_0_ and therefore the unit of *κ* is µm^−1^ [41], [55].

We simulated this model using the same value of *γ*∼0.4 stated in the previous section. Mean position of the KT and its fluctuation around the mean value for other values of *γ* are shown in Figure S9 in File S1. As the kMT penetrates deeper into the KT cylinder, it continues to push the KT away from the SPB. However, due to the length dependent catastrophe, the kMT cannot grow too long and eventually switches into the depolymerization state. The unconstrained depolymerization of the kMT is resisted by the couplers (Figure 5A) applying tension on the kMT whenever the latter attempts to leave the KT cylinder. This enhances the rescue probability of the kMT (Equation 3) allowing it to remain within the KT cylinder.

Simulated distance of the KT from the SPB using anaphase parameters is plotted against time in Figure 5B for both wild-type and mutant cells. Mean KT distance from the SPB is *∼*350±250 nm for the wild-type and ∼460±290 nm for the mutant. Data obtained using G1 parameters (see Figure 5C) predict the mean KT distance as ∼440±290 nm and ∼580±320 nm for the wild-type and mutant respectively. All our simulation results are in reasonable agreement with the experimental data.

In *chl4* and/or *ctf19* strain, we assumed that the modified protein configuration within the KT stiffens the effective coupling strength between kMT and KT. This idea is incorporated in the model by increasing the force-constant *κ* of the couplers. Stiffer coupling enhances the tension on the kMT facilitating frequent rescue of the latter. Thus compared to the wild-type, in mutant cells kMT remains in the growth phase more often than in the shrinking phase, leading to increased distance between the SPB and KT. In the present simulation we did not allow KTs to detach from the kMT. However, if the applied tension on the kMT-KT couplers exceeds a certain threshold, KTs may actually detach from the kMT leading to chromosome loss. Data obtained from the simulation is shown in Figure S10 in File S1 [35].

## Discussion

In this work we have investigated the potential role of Chl4p and Ctf19p, the non-essential KT proteins of the Ctf19 complex, in centromere/KT positioning. We have found that in the absence of these proteins, KT positioning is affected in G1 and in late anaphase. We also report that a spindle pole body protein, Bbp1, associates physically with Ctf19p and Chl4p by using yeast two-hybrid assays and in vitro GST pull-down experiments. However, we have shown that the SPB-centromere proximity is maintained by MTs only, and is not dependent on physical interactions between SPB and KT components, even partially, as suggested by earlier studies [59]. We have further elaborated on the possible significance of physical interactions between SPB and KT components in the supplementary section (Text S6 in File S1). Combined cell biology and computer generated simulation studies suggest that the proximity between SPB and centromere/KT depends on KT integrity and MTs, and is regulated by MT-dependent forces. In contrast to the budding yeast, centromere clustering observed in interphase cells of the fission yeast *Schizosaccharomyces pombe* is independent of MTs and depends on protein-protein interactions [60], [61], [62]. The centromeres of this yeast are clustered at the nuclear envelope in close vicinity to the cytoplasmic SPB [63]. In a recent study, a protein Csi1 has been identified that localizes to the centromeres of interphase chromosomes and links the centromeres to the nuclear envelope by interacting physically with a nuclear envelope protein found concentrated at a site close to the SPB [62]. Csi1p could also act as a molecular linker between interphase centromeres and SPB via nuclear envelope and SPB proteins. In the budding yeast, apart from a brief interval during DNA replication, the centromeres stay tethered to kMTs throughout the cell cycle and show constrained motion towards and away from the attached SPBs [14], [18]. As explained in Text S6 in File S1, a direct centromere to SPB contact is precluded in this scenario. Our results further show that despite the documentation of several protein-protein interactions between SPB and kinetochore components in this yeast (Table S2 in File S1), SPB and centromeres do not establish proximity to form stable attachments in the absence of MTs. Therefore, the mechanism by which centromeres of interphase fission yeast cells cluster near the SPB is unlikely to be operative in the budding yeast.

The general principle of the positioning of a KT attached to a MT is described by the dynamical characteristics of the latter, which would be influenced by the attached load, the KT itself. Both Chl4p and Ctf19p affect KT structure and its interaction with the MT, as determined by stabilization of the dicentric minichromosomes [25], [37], sensitivity to MT network disrupting drug benomyl [8], [35], reduced MT binding by *ctf19* cells to minichromosomes in an *in vitro* assay [35] and ease of transcription through KT in the mutant cells belonging to the Ctf19 complex [23], [24]. This last feature suggests that the KT is less compact in Ctf19 complex mutants. Therefore, we reasoned that the KT would experience altered MT-based forces in these mutants which, in turn, could alter their positions relative to the SPBs within the nucleus. The three dimensional distance measurement between spindle pole and centromere (*CEN5*) in the fixed late anaphase cells of wild-type, *chl4*, *ctf19* and *chl4 ctf19* revealed that, on an average, SPB-*CEN5* distance in mutant anaphase and G1 cells was increased by 20–40% over that in the corresponding wild-type cells (Figures 1E and 3, and Figures S4 and S5 in File S1).

How might Ctf19p and/or Chl4p affect the KT positioning? Ctf19 complex is necessary for the initial capture of the KT by the lateral surface of the MT, following a brief period of detachment in S-phase [11], [12]. KT is transported towards the SPB by two ways: lateral sliding and end-on pulling, with lateral sliding often converting into the end-on pulling [12], [64]. The Dam1 complex, specifically Dam1p, is crucial for the end-on pulling force towards SPB on the KT [64]. It has been shown previously that purified Chl4p and Ctf19p directly bind to Dam1p *in vitro* and a two-hybrid interaction between Ctf19p and Dam1p is also reported [65], [66]. In the absence of Chl4p and Iml3p, (the latter being a partner protein of Chl4p in a subcomplex), the two-hybrid interaction between Ctf19p and Dam1p is severely weakened or lost [66]. Additionally, in the *iml3* mutant, Dam1p molecules show a slight reduction in centromere localization [66]. Since Iml3p depends upon Ctf19p for centromere localization, it can be argued that in the absence of Ctf19p, the localization of Dam1p at the centromere is somewhat impaired [8]. As Dam1p plays a crucial role for the end-on attachment of the KT, the disruption of native Dam1p organization is expected to affect pulling forces on the KT. Therefore, conceivable reasons of increased separation between SPB and KT in *chl4* and/or *ctf19* could be two-fold: One, a compromised end-on pulling action of the Dam1p on KT, restricting it to distances further away from the SPBs and two, structural alterations in the mutant KT make it less compact (see above) and offer lesser resistance to a growing MT.

We have also carried out computer simulations and developed models, based on length and load-dependent MT dynamics, that support our results on SPB-*CEN* distances in wild-type and mutant cells. Experiments [50], [52] and computational models [49] predict that frequencies at which MTs grow and shorten are highly affected by mechanical constraints such as the applied load and the length of the MT. The tip of the kMT embedded inside the KT mechanically couples with the resident proteins and thus evolves differently in wild-type and mutant KTs. Several computational models based on polar ejection force, unconstrained and constrained kMT dynamics have been explored in order to understand the possible cause for the increased distance between SPB and KT. In both these scenarios, the position of the KT fluctuates widely and often become unrealistic, if unconstrained kMT dynamics (Figure S6 in File S1) or polar ejection forces (Figure S7 in File S1) are used. Thus we propose a third scenario based on the altered kMT dynamics, viz regulation of catastrophe and rescue frequencies. The length and force dependent regulation of kMT dynamics can independently confirm the observed experimental results satisfactorily [49]. In the two variants, both catastrophe and rescue frequencies are assumed to be modified by the applied load on the kMT. Essentially, the magnitude of the resistance offered by the KT plate or the KT fibrils while kMT is growing or shrinking within the KT cylinder respectively depends upon the instantaneous length of the kMT. These models suggest that the absence of Chl4p and/or Ctf19p, the components of Ctf19 complex possibly modifies the protein configuration within the KT cylinder facilitating growth or enhancing the rescue frequency of the kMT.

Although our computational model consistently reproduces qualitative features of the KT positioning in the wild-type and in the mutant yeast, simulated distances between SPBs and the KTs appeared to deviate from the values obtained experimentally for G1 cells. For example, for wild-type G1 α-factor arrested cells, the experimental value of 730 nm was higher than the simulated values of 510 and 440 nm, derived respectively from the regulation of catastrophe and rescue frequency models. However, wild-type G1 cells from an asynchronous culture gave an experimental value of 570 nm (Figure S5 in File S1), which was much closer to the simulated values described above. We believe that G1 cells from the asynchronous culture are free from α-factor-mediated effects, if any, on MT dynamics and represent a more realistic picture [18]. The observed deviation between the experimental and simulated values could arise due to i) the lack of a more sophisticated model and/or ii) imprecise model parameters used for the G1 phase (Table 1). The model proposed here is drastically simplified by coarse-graining molecular interactions between MT and Ctf19 complex. The mechanical interaction between MT and various resident proteins of the KT is no doubt, far more complex than a simple linear spring, as assumed in the model. Thus our study leaves a great opportunity to develop a detailed model built with individual KT proteins and a realistic MT assembled from individual tubulin monomers. Considering the fact that KT position correlates directly with the MT dynamics, our *in silico* results were found to be sensitive to the dynamical parameters used in this study.

This work shows that, apart from compromising fidelity of chromosome transmission, the Ctf19 complex also regulates the localization of centromeres within the nucleus. The positioning of centromere during mitosis can be crucial for proper chromosome segregation. The reconstitution of the yeast KT from its purified subcomplexes would go a long way in determining the contribution of its various subcomplexes towards MT-derived forces it encounters. Such studies can give a more detailed picture of chromosome movement during mitosis.

## Supporting Information

File S1 **Contains the following files: Text S1. Additional methodologies. Text S2. Chl4p and Ctf19p show two-hybrid interactions with Bbp1p. Text S3.** ***In vitro*** **experiments confirm the association of Bbp1p with Ctf19 and Chl4 proteins. Text S4. Unconstrained kinetochore microtubule (kMT) dynamics cannot predict KT position. Text S5. Competing polar ejection force and kMT tension predicts mean KT position but does not qualify the coefficient of viscous drag. Text S6. Physical interactions between SPB and KT components: possible significance. Figure S1. Physical interaction of Chl4p and Ctf19p with Bbp1p.** (A) Physical interactions of Bbp1p with Chl4 and Ctf19 proteins. PJ69-4A was transformed with two-hybrid plasmids pGAD424 and pGBT9, the former expressing partial (clones **a** to **d**) or full length Bbp1p ORF fused to the *GAL4* activation domain, and the latter expressing Chl4p or Ctf19p fused to the *GAL4* binding domain. The transformants were selected on SC plates lacking leucine and tryptophan (-Leu-Trp). Freshly growing cells from these plates were streaked for growth at 30^ο^C on -Leu-Trp and SC plates lacking leucine, tryptophan and histidine (-Leu-Trp-His). Transformants growing on -Leu-Trp-His plates showed two-hybrid interactions between the fused proteins as described under methods. (B) Schematic diagram depicting different fragments of Bbp1p which interacted with Chl4 and Ctf19 proteins in two-hybrid screening. All the fragments contained two coiled-coil (c.c) domains at amino acids 235–275 and 311–359. +, two-hybrid interaction; -, no two-hybrid interaction. (C) Physical interaction of Mcm22p with Bbp1p. The PJ69-4A strain was transformed with two-hybrid plasmids expressing AD-Bbp1p and BD- Mcm22p. Other details were as described above. **Figure S2. Physical association of Chl4p and Ctf19p with Bbp1p** ***in vitro*** **.** (A) Purification of Bbp1p expressed in *E. coli.* Cell extracts were isolated from *E. coli* strain BL21 (DE3) carrying pGEX-5X-2 (vector) and pGEX-5X-2-*BBP1* and purified using glutathione Sepharose beads. Bound proteins were eluted with SDS-sample buffer and fractioned on a 10% polyacrylamide gel and silver stained. Lanes 1 and 2 are eluted GST-Bbp1p and GST fusion proteins, respectively. GST-Bbp1p fusion should migrate at 72 kD. (B) Immunoblot of Bbp1p using anti-GST antibody. The eluted proteins from Figure 2A were loaded in 10% polyacrylamide gel and immunoblotted using anti-GST antibody. Lanes 1 and 2 are purified GST and GST-Bbp1p fusion proteins, respectively. Protein size markers (kD) are also shown on the side. (C and D) *In vitro* association of Chl4p and Ctf19p with Bbp1p. AP22 (wild-type) strain was transformed separately with pACT2 expressing *HA*, *HA-CHL4* or *HA-CTF19* and GST pull-down studies were carried out as detailed in Materials and methods, by mixing cell extracts obtained from each transformant with equal amounts of purified bead-bound GST or GST-Bbp1p. Proteins eluted from glutathione Sepharose beads were loaded on 10% polyacrylamide gels for Western blotting, as described under methods. (C) Anti-GST immunoblot, for detecting GST-Bbp1p and GST proteins in GST pull-down fractions. (D) Anti-HA immunoblot, for detecting HA, HA-Chl4p and HA-Ctf19p in GST pull-down fractions. Lanes 1 and 6 show protein bands corresponding to HA-Chl4p (72 kD) and HA-Ctf19p (62 kD), respectively, in the cell extracts of the corresponding transformants. Asterix indicates a non-specific band that tends to appear in lanes carrying GST-Bbp1p when probed with anti-HA. **Figure S3. Wild-type centromeres lie closer to SPBs than mutant centromeres upon MT disruption by nocodazole.** Exponentially growing cells of US3329 (wild-type) and US3329Δctf19Dchl4 (*chl4 ctf19*) in YEPD at 30^ο^C were arrested at G2/M using nocodazole (15 µg/ml) for 120 minutes. Cells were fixed with formaldehyde and anti-α-tubulin was used to stain SPB, which appeared as a round spot in about 20-30% of the cells. Images were captured in 0.5 µm z-sections and analyzed by confocal microscopy. (A) Upper panel: A section of z-stack is shown containing three large budded cells with SPBs visible as red dots, but with no visible spindles. In the same section, one cell also shows a *CEN5*-GFP which is co-localized with SPB (yellow spot in merged figure), another cell shows non-overlapping SPB and *CEN5*-GFP dots and the third cell has its *CEN5*-GFP dot in the next section of z-stack (not shown). Middle and lower panels: Cells are shown with well-separated *CEN5*-GFP and SPB dots. The horizontal scale bar represents 1 µm. (B) The bar diagram represents percentages of cells in which the 3D distance between *CEN5* and SPB lies within 300 nm. **Figure S4.** ***ctf19*** **anaphase cells without nocodazole treatment show increased SPB-KT distance over corresponding wild-type cells.** Exponentially growing wild-type *CTF19* (PS1) and mutant *ctf19* (PS1Δctf19) cells were arrested in G1 by α-factor and, after 145 minutes of release from the arrest, they were harvested and treated for microscopy as described earlier (Materials and methods). The synchronization with α-factor increased the proportion of anaphase B cells in the culture. SPB-KT distances were measured as described in Materials and methods for this figure. (A) Cells showing the separation of SPBs (red) from their centromeres (green). Upper panel, wild-type cells; lower panel, mutant *ctf19* cells. Scale bars, 2 µm. (B) The histogram shows the distribution of centromeres based on their distances from their SPBs. The average distances for the wild-type and the mutant strains were 620±241 and 710±349 nm, respectively. 65 centromeres were analysed from the wild-type and 66 from the mutant strain. Note: The fraction of wild-type kinetochores lying within 500 nm of their SPBs was about 1.7 times the corresponding fraction of mutant kinetochores, which is similar to that obtained for nocodazole-treated cells (Figure 1B). However, the absolute values of SPB-KT distances for both the strains are higher than those obtained for nocodazole-treated cells (Figure 1). We believe that this could be due to one or both of the following reasons. One, the Spc110p-RFP tag used here for locating the SPB is at the C-terminus of the protein which lies at the central plaque of the SPB, about 100 nm away from the inner plaque [38]. In the earlier experiment (Figure 1) SPB was identified by α-tubulin antibody which stains the inner plaque within the nucleus, thereby increasing the SPB-KT distance by about 100 nm in the present experiment over the earlier one. The second reason could be the use of a different microscope and its software for measuring distances in the present experiment, since RFP fluorescence could not be detected satisfactorily by the confocal microscope used in the earlier experiment. **Figure S5. G1 cells of asynchronous** ***ctf19*** **culture show increased SPB-KT distance compared to corresponding wild-type cells.** Exponentially growing cells of *CTF19* (PS1) and *ctf19* (PS1Δctf19) strains were treated for fluorescence microscopy (Materials and methods). G1 cells were identified as being round and unbudded. SPB-KT distances were measured as described in Materials and methods for this figure. (A) G1 cells illustrating separation betweenSPB (red) and centromere (green) in wild-type (upper panel) and mutant (lower panel) cells. Scale bars, 2 µm. (B) Scatter plots showing the distance of each centromere from its SPB. 36 cells were analysed in each case. The average distances were 570±211 nm (wild-type) and 709±244 nm (mutant). **Figure S6. Unconstrained MT dynamics derived KT positioning.** (A) Schematic of the model showing a KT attached with a single MT moves toward and away from the SPB (pole) coherently with the growth and shortening of the kMT. Three different positions of the KT are shown. (B) Probability distribution of the KT position measured from the SPB for anaphase and G1. Inset shows the time dependent trajectory of a KT. **Figure S7. Competing ‘polar ejection force’ and kMT tension requires high viscosity to predict mean KT position.** (A) Working model: Blue sphere represents the SPB and the green rods emerging from the SPB depict MTs. MTs generate ‘polar ejection force’ as they impinge upon the chromosome arm (light green structure). This force is enhanced by plus end directed chromokinesin motors acting at the overlap between MT and chromosome arm. Red arrows show the direction of the poleward tension between KT and SPB. (B) Position of the KT, measured from the SPB, is plotted as a function of time for different values of the viscous drag coefficient (ς). A large spatial fluctuation (∼±300 nm) about the mean KT position is observed for ς ∼5 pN-µm/s. Such fluctuations can be reduced only if a very large value of the ς ∼100 pN-µm/s is used in the simulation. **Figure S8. Mean KT position; fluctuation increases with efficiency (γ) of the KT being pushed by kMT.** (A) Mean position of the KT (X_KT_) measured from SPB is plotted against γ increases for both WT and mutant cells. For small γ, mutant KT position is further from the SPB than the wild-type. Increasing γ effectively stiffens the linker configuration amplifying the catastrophe frequency of the MT. For γ close to unity the linker proteins do not distinguish between a WT and mutant and therefore the difference between the mean KT positions also vanishes. (B) Fluctuation in the mean KT position [measured from the full width at half maxima (FWHM) of the Gaussian fits] increases with γ. **Figure S9. Mean KT position; fluctuation increases with efficiency (γ) of the KT being pulled by kMT.** (A) Mean position of the KT (X_KT_) measured from SPB is plotted against γ decreases for both WT and mutant cells. Difference in the KT position between wild type and mutant is reduced for large values of γ. In the limit γ close to unity the linker proteins do not distinguish between a WT and mutant and therefore the difference between the mean KT positions also vanishes. (B) Fluctuation in the mean KT position [measured from the full width at half maxima (FWHM) of the Gaussian fits] increases with γ. **Figure S10. Chromosome loss statistics.** Percentage of chromosome detached from the kMT detachment force. KT detaches from kMT once the tension between them exceeds a threshold. Data shown here is obtained from a simulation over 100000 samples. We find that in the wild-type (WT) cell, ∼0.03% chromosome is lost for a threshold tension ∼8.85 pN. For the same magnitude of threshold, chromosome loss for the mutant cell is ∼16%. Our model prediction for the WT cell agrees with the experimental results [19]. **Table S1. Strains used in this study. Table S2. Physical interactions between KT and SPB proteins. Table S3. Model parameters.**
(DOCX)
