# Supplementary material for: Budding Yeast Kinetochore Proteins, Chl4 and Ctf19, Are Required to Maintain SPB-Centromere Proximity during G1 and Late Anaphase
Source: PLoS One. 2014 Jul 8;9(7):e101294. doi: 10.1371/journal.pone.0101294 (PMC4086815; doi:10.1371/journal.pone.0101294)
Supplement: File S1 — Contains the following files: Text S1. Additional methodologies. Text S2. Chl4p and Ctf19p show two-hybrid interactions with Bbp1p. Text S3. In vitro experiments confirm the association of Bbp1p with Ctf19 and Chl4 proteins. Text S4. Unconstrained kinetochore microtubule (kMT) dynamics cannot predict KT position. Text S5. Competing polar ejection force and kMT tension predicts mean KT position but does not qualify the coefficient of viscous drag. Text S6. Physical interactions between SPB and KT components: possible significance. Figure S1. Physical interaction of Chl4p and Ctf19p with Bbp1p. (A) Physical interactions of Bbp1p with Chl4 and Ctf19 proteins. PJ69-4A was transformed with two-hybrid plasmids pGAD424 and pGBT9, the former expressing partial (clones a to d) or full length Bbp1p ORF fused to the GAL4 activation domain, and the latter expressing Chl4p or Ctf19p fused to the GAL4 binding domain. The transformants were selected on SC plates lacking leucine and tryptophan (-Leu-Trp). Freshly growing cells from these plates were streaked for growth at 30οC on -Leu-Trp and SC plates lacking leucine, tryptophan and histidine (-Leu-Trp-His). Transformants growing on -Leu-Trp-His plates showed two-hybrid interactions between the fused proteins as described under methods. (B) Schematic diagram depicting different fragments of Bbp1p which interacted with Chl4 and Ctf19 proteins in two-hybrid screening. All the fragments contained two coiled-coil (c.c) domains at amino acids 235–275 and 311–359. +, two-hybrid interaction; -, no two-hybrid interaction. (C) Physical interaction of Mcm22p with Bbp1p. The PJ69-4A strain was transformed with two-hybrid plasmids expressing AD-Bbp1p and BD- Mcm22p. Other details were as described above. Figure S2. Physical association of Chl4p and Ctf19p with Bbp1p in vitro . (A) Purification of Bbp1p expressed in E. coli. Cell extracts were isolated from E. coli strain BL21 (DE3) carrying pGEX-5X-2 (vector) and pGEX-5X-2-BBP1 and purified u [file pone.0101294.s001.docx]

**Manuscript number:** PONE-D-14-07107R1

**Revised date:** June15, 2014

# Supporting Information

# Text S1. Additional methodologies

***Two-hybrid studies***

The plasmids pGAD424 and pGBT9 were used for two-hybrid studies. *BBP1* ORF was fused in frame to the *GAL4* DNA activation domain (AD) of *GAL4* in pGAD424 to get AD-*BBP1*. The ORF of the kinetochore (KT) protein under study was fused to the *GAL4* DNA binding domain (BD) in pGBT9. *BBP1* ORF fusion to the DNA binding domain in pGBT9 activated gene expression even in the absence of pGAD424, so that protein-protein interactions could not be studied using BD-*BBP1*. The plasmids pGAD424 and pGBT9, with or without gene fusions, were transformed into the strain PJ69-4A. Freshly growing transformants from synthetic complete (SC) plates lacking leucine and tryptophan (SC-Leu-Trp) were 10-fold serially diluted and spotted on control permissive plates SC-Leu-Trp, and on plates selective for growth only in the presence of two-hybrid interactions between the fused proteins [[1](#_ENREF_1)]. These were SC plates lacking leucine, tryptophan and histidine (SC-Leu-Trp-His), SC plates lacking leucine, tryptophan, histidine and containing 2.5 mM 3-aminotriazole (3-AT) that was added to increase the stringency of selection in the absence of histidine (SC-Leu-Trp-His + 2.5 mM 3-AT) and SC plates lacking leucine, tryptophan and adenine (SC-Leu-Trp-Ade). Interaction between two proteins, say X and Y, was assumed to have occurred if the transformant carrying both BD-X and AD-Y could grow on selective plates.

***GST pull-down assay***

To express *BBP1* in *Escherichia coli*, *GST-BBP1* was generated (see Text S1). For *GST* and *GST-BBP1* expression, *E. coli* BL21 (DE3) strain was transformed with *GST* (pGEX-5X-2) and *GST-BBP1* (pGEX-5X-2-*BBP1*). Transformants carrying these plasmids were grown to an A_595_ of 0.5. Isopropyl-*β*-D-thiogalactopyranoside (IPTG) was added to a final concentration of 1 mM and growth was continued at 37^ο^C with shaking for 3 hours. Cells were centrifuged and resuspended in PBS buffer (pH 7.4) plus 5% glycerol and 1 mM phenylmethylsulfonylfluoride (PMSF). The resuspended cells were subjected to sonication and then centrifugation (12000 r.p.m. for 30 minutes at 4^ο^C in SS34 Sorvall rotor) to obtain clear cell lysate. Protein concentration was measured by Bradford method [[2](#_ENREF_2)]. GST and GST-Bbp1p present in supernatants were taken in equal amounts (~7 mg each) and to each an equal volume of 50% slurry (v/v) of glutathione-Sepharose 4B beads (Amersham Biosciences) was added along with Triton X-100 (1% final concentration) and rocked for 1 hour at 4^ο^C. The beads were washed once with PBS buffer containing 0.1% Triton-X-100 and twice with PBS buffer alone. To remove bound proteins, beads were mixed with SDS-sample buffer, suspended for 10 minutes in a boiling water bath. The eluted proteins were loaded in equal amounts and run in duplicate 10% SDS-polyacrylamide gels. One gel was silver stained [[3](#_ENREF_3)] while with the other a Western blot was performed as described in reference [[4](#_ENREF_4)], except for a change in acrylamide and bis-acrylamide ratio which was kept as 29:1. Densitometric analysis of the silver stained gel was done using Molecular Analyst Software (Bio-Rad). A band corresponding to GST-Bbp1p appeared at the expected size in the silver stained gel and showed some degradation products (Figure 2A). The amount of GST produced was normally found to be about 10-fold higher as compared to GST-Bbp1p, including its degraded products (Figure 2A). The immunoblot, visualized using mouse monoclonal anti-GST antibody (1:750 dilution, Santa Cruz Biotechnology, Inc.) and goat anti-mouse alkaline phosphatase (1:2500 dilution, Sigma), revealed protein bands (GST and GST-Bbp1p) of the correct molecular weights (Figure 2B).

For *in vitro* physical interaction studies, *E. coli* BL21 (DE3) cell extracts carrying GST and GST-Bbp1p were prepared as described above. Since the expression of GST was roughly 10-fold higher than that of GST-Bbp1p, the bacterial cell extract containing GST was 10-fold lower than that of GST-Bbp1p. Bead bound proteins were prepared as described above. Log phase yeast cells containing HA-Ctf19p, HA-Chl4p, HA-Chl4p *iml3*∆ and HA alone were pelleted and washed with 0.9% NaCl. The pellet was resuspended in L-buffer [50 mM HEPES-HCl (pH 7.4), 100 mM NaCl, 1 mM EDTA (pH 8.0), 1 mM PMSF, 5 % glycerol, 1μg/ml Pepstatin A, 0.5 μg/ml Leupeptin, 1 μg/ml Aprotinin and protease cocktail (Sigma)], sonicated and centrifuged at 12,000 r.p.m. for 30 minutes at 4^ο^C. Equal amounts of protein from each cell lysate (5 mg) were incubated separately with equal amounts of bead bound GST and GST-Bbp1p and rocked for 45 minutes at 4^ο^C. The matrix was sedimented by centrifuging at 3000 r.p.m. for 3 minutes at 4^ο^C and washed four times with ten bed volumes of wash buffer [50 mM HEPES-HCl (pH 7.4), 100 mM NaCl, 1 mM EDTA (pH 8.0) and protease cocktail]. Bead bound proteins were removed using SDS-sample buffer and Western blot was performed. The HA epitope was visualized using rat monoclonal anti-HA 3F10 antibody (1:500 dilution, Roche Applied Science) and rabbit anti-rat alkaline phosphatase (1:2500 dilution, Sigma).

#

***Gene deletions***

*IML3* deletion was carried out in PJ69-4A∆chl4 using the *E. coli kan^r^* gene which confers resistance to G418. The plasmid pM31-3 [[5](#_ENREF_5)] is disrupted-deleted by a *URA3*-carrying *Sma*I-*Sal*I fragment [[5](#_ENREF_5)]. This fragment carries an internal *Bam*HI site. A 2.5 kb *Bam*HI-*Sal*I fragment carrying *LEU2* gene from pL2 [[6](#_ENREF_6)], having an internal *Hpa*I site, was cloned at the *Bam*HI-*Sal*I sites of pM31-3. The resulting plasmid was termed as pM31-4. The *LEU2* gene of pM31-4 was replaced by cloning a 1.5 kb *Sal*I-*Eco*RV fragment containing *kanMX6* module from pFA6a-kanMX6 [[7](#_ENREF_7)] at the *Sal*I-*Hpa*I sites of pM31-4 to obtain pM31-5. In this plasmid, *IML3* gene was deleted-disrupted by *URA3*- *kanMX6* construct. The *Pvu*II fragment of pM31-5 was used to delete *IML3*. *CTF19* deletions in PJ69-4A and US3329 were done as follows. A 2.6 kb long *Xba*I fragment containing the *CTF19* open reading frame (ORF) was subcloned into the *Xba*I site of YIplac211 [[8](#_ENREF_8)], having its *Eco*RI site destroyed, termed as YIplac211-2. To create a deletion of *CTF19*, a 1.4 kb *Eco*RI-*Bam*HI fragment of pUC19U [[9](#_ENREF_9)] carrying *URA3* gene was cloned into the *Eco*RI-*Bgl*II sites of the YIplac211-2, termed as YIplac211-3. The 3 kb *Xba*I fragment of YIplac211-3 carrying the deleted ORF was used for deleting *CTF19*.

***Construction of two-hybrid plasmids***

For two-hybrid studies, pGBT9 [[10](#_ENREF_10)] carrying the *GAL4* DNA binding domain (BD) and pGAD424 [[10](#_ENREF_10)] carrying the *GAL4* activation domain (AD) were used. To construct AD*-BBP1* and BD*-BBP1*, amplification of *BBP1* was done using the forward primer

5′-CGGGATCCCGATGAATCAGGAAGACAACACGGGCGG-3' which introduced the *Bam*HI site (underlined) and also contained the coding sequence beginning with ATG, and the reverse primer 5'-GGCAGGACTACCAATTAGGCCCGG-3', upstream of which contained an endogenous *Pst*I site. The ~1.8 kb PCR product was digested with *Bam*HI and *Pst*I and this *Bam*HI-*Pst*I fragment was ligated to *Bam*HI-*Pst*I digested pGAD424 and pGBT9 to get AD-*BBP1* and BD-*BBP1*, respectively. The constructions of BD-*MCM16*, BD-*CTF19*, BD-*MCM21* and BD-*MCM22* were done according to [[11](#_ENREF_11)] and are available upon request. BD-*CHL4* and BD-*IML3* are described in [[5](#_ENREF_5)].

***Epitope tagging***

For GST pull-down assays *CHL4* and *CTF19* were HA-tagged N-terminally. BD-*CHL4* and BD-*CTF19* plasmids [[5](#_ENREF_5),[11](#_ENREF_11)] were digested with *Bam*HI and *Sal*I. Both digestions yielded 1.7-kb fragments and were ligated separately to *Bam*HI-*Xho*I [present at multiple cloning site (MCS)] digested pACT2 [[12](#_ENREF_12)] to obtain pACT-*CHL4* and pACT-*CTF19* constructs. pACT2 plasmid contains an HA-epitope before its MCS. In frame cloning of *CHL4* and *CTF19* at the MCS of pACT2 plasmid generated N-terminal HA tagging, termed as HA-*CHL4* and HA-*CTF19*, respectively.

C-terminal tagging of chromosomal *BBP1* with thirteen tandem repeats of Myc epitope was done according to [[13](#_ENREF_13)]. For this, a 2169 bp PCR product was obtained using pFA6a-13Myc-His3MX6 plasmid as template and the following forward and reverse primers, respectively: 5'- GTAATAACGAGAGAAAAGATACTTCTGCTGGTTCGAATATTTTTTCAACAGGACAA CGGATCCCCGGGTTAATTAA-3' and 5'- GAATGCGAATTAGAGTCGTAACTGTTATTTCCATTGTGGAATGGAGTCCTGTC GAATTCGAGCTCGTTTAAAC-3'. The underlined 56 and 53 nucleotides are immediate upstream (forward primer) and downstream (reverse primer) of *BBP1* stop codon. The amplified DNA was transformed directly into wild-type yeast strain AP22 and transformants carrying *BBP1* tagged at its C-terminal end with 13Myc were selected on SC plates lacking histidine. Chromosomally tagged *BBP1-13MYC* gene fusion was checked by PCR and protein expression was checked by Western blot using anti c-Myc 9E10 antibody (Santa Cruz Biotechnology, Inc.).

To express *BBP1* in *Escherichia coli*, *GST-BBP1* was generated. To construct *GST-BBP1*, AD-*BBP1* was digested with *Bam*HI and *Pst*I and complete *BBP1* ORF was obtained. This fragment was cloned at *Bam*HI –*Pst*I sites of pMV261 [[14](#_ENREF_14)], termed as pMV261-*BBP1*. This plasmid was digested with *Bam*HI- *Sal*I and complete *BBP1* ORF was inserted in frame at *Bam*HI- *Sal*I digested pGEX-5X-2 [contains glutathione S-transferase (GST) before MCS] plasmid. The resultant plasmid termed as *GST-BBP1* where GST was at the N-terminal of *BBP1* ORF.

**Text S2. Chl4p and Ctf19p show two-hybrid interactions with Bbp1p**

To search for additional members of the KT complex, Chl4p was used as the bait protein in the *GAL4*-based two-hybrid system. *GAL4* activation domain fusion genomic libraries [[1](#_ENREF_1)] were used to identify novel interactors of this protein. Four interacting clones were obtained and sequence analysis showed that each of them carried a part of an essential spindle pole body component *BBP1* [[15](#_ENREF_15)]. These four clones were also tested for two-hybrid interaction with several other KT proteins of the Ctf19 complex, namely Mcm16, Ctf19, Iml3, Mcm21 and Mcm22 [[16](#_ENREF_16),[17](#_ENREF_17),[18](#_ENREF_18),[19](#_ENREF_19)]. Of the four interacting clones, three (**a, b, c**) were identical in that each carried amino acids 198-385 of full length Bbp1p (385 amino acids) and interacted only with Chl4p (shown for interactor **a** in Figure S1, A upper panel and B). The clone **d** contained amino acids 151-385 and, in addition to Chl4p, also interacted with Ctf19p (Figure S1, A and B). *BBP1* open reading frame (ORF) was fused to the *GAL4* activation domain (AD) and binding domain (BD). When fused to the binding domain, the plasmid *GAL4* (*BD*)-*BBP1* along with the control vector *GAL4* (AD), activated the reporter genes and could not be used for testing interactions with other proteins. Therefore, only the *GAL4* (*AD*)-*BBP1* construct was used to test the interaction of this protein with some of other KT proteins (Mcm16, Chl4, Ctf19, Iml3, Mcm21, Mcm22 and Duo1). Robust interaction was obtained only with Chl4p and Ctf19p (Figure S1A). The sequence of Bbp1p from 198-385 amino acids contains both its coiled coil domains, which are required for the interaction of Bbp1p with another spindle pole body (SPB) protein Mps1p.

It has been reported that Mcm22p gives two-hybrid interactions with Bbp1p [[20](#_ENREF_20)]. We found that transformants carrying AD-*BBP1* and BD-*MCM22* grew weakly, if at all, on plates selective for two-hybrid interactions. This suggests that Bbp1p and Mcm22p interact only weakly with each other (Figure S1C).

**Text S3. *In vitro* experiments confirm the association of Bbp1p with Ctf19 and Chl4 proteins**

To confirm the two-hybrid interactions of Bbp1p with the KT proteins Chl4p and Ctf19p, co-immunoprecipitation studies were carried out. The chromosomal copy of *BBP1* was tagged with the 13X Myc epitope (see Text S1). Chl4 and Ctf19 were each tagged with HA by expressing the corresponding genes from pACT2 plasmid (see Text S1). Bbp1-Myc protein was pulled down from cell-free extracts of transformants carrying Chl4-HA (or Ctf19-HA) using anti-Myc antibodies. The immunoprecipitate was resolved on an SDS-polyacrylamide gel and the Western blot was probed with anti-HA antibodies to detect Bbp1p two-hybrid binding partners Chl4p and Ctf19p. However, even after several attempts that involved changing of conditions of cell lysis, we were unable to detect either Chl4p or Ctf19p on the Western blot of the immunoprecipitate. Similar observations have been made for Bbp1p and Spc29p, where the two proteins gave two-hybrid interactions but could not be co-immunoprecipitated together. However, *in vitro* GST pull-down experiments did confirm their association [[21](#_ENREF_21)]. We used the same strategy to detect the interactions of Bbp1p with Chl4 and Ctf19 proteins. For this, the entire *BBP1* ORF was cloned in frame with GST in pGEX-5X-2 vector to get *GST-BBP1* and the fusion protein was expressed in *E. coli* BL21 (DE3) strain. The GST-tagged protein was purified using glutathione Sepharose beads and the identity of GST-Bbp1p was confirmed by silver staining and immunoblotting with anti-GST antibody (Figure S2, A and B). GST-Bbp1p appeared to be unstable in that several degradation products appeared on the gel despite the presence of recommended amounts of protease inhibitors. Glutathione Sepharose beads coupled to equal amounts of GST or GST-Bbp1p, including its degradation products (see Text S1), were incubated with equal amounts of proteins from cell extracts of transformants containing HA-Ctf19p, HA-Chl4p (each cloned on the plasmid pACT2) or HA (vector pACT2 alone). Western blots of the eluted proteins were done using anti-GST and anti-HA antibodies (Figure S2, C and D). Cell extracts of transformants containing HA-Chl4p, HA-Ctf19p and HA alone (vector control) were also loaded in the gel to identify the bands corresponding to HA-Chl4p and HA-Ctf19p. The immunoblots revealed that HA-Chl4p and HA-Ctf19p specifically associated with only GST-Bbp1p, but not with GST alone (Figure S2D**,** lanes 2, 3 and 7, 8). The low intensity of the immunoprecipitated Chl4p band could be due to lower level of affinity between Bbp1p and Chl4p. Lanes 1 and 6 (Figure S2D) show positions of HA- Chl4p and HA-Ctf19p, respectively. As expected, no band corresponding to either of these fusion proteins was obtained in lanes where cell extracts from transformants carrying the vector alone were loaded (Figure S2D, lanes 4 and 9). Also, GST-Bbp1p did not associate with HA alone (Figure S2D, lanes 5 and 10). In Western blots of anti-HA (Figure S2D), a non-specific band appeared (marked with an asterix) in lanes which carried GST-Bbp1p protein extracts. The origin of this band is not clear. It is possible that GST-Bbp1p (or one of its degradation products) was binding to some unknown protein that was recognized by the anti-HA antibody.

**Text S4. Unconstrained kinetochore microtubule (kMT) dynamics cannot predict KT position**

We started with a model in which the position of the KT is determined by the trajectory of the plus end of a dynamic kMT (see Figure S6A). MTs are protein filaments, which grow and shrink stochastically [[22](#_ENREF_22)]. Due to a mechanism termed dynamic instability, MTs switch between catastrophe (growth to shortening) and rescue (shortening to growth) events [[23](#_ENREF_23),[24](#_ENREF_24)]. The dynamics of a MT can be interpreted by four parameters, viz., growth velocity (*v*_0_), shrink velocity (*v*_s_), catastrophe frequency (*f*_c_^0^) and rescue frequency (*f*_r_^0^).

In this approach no constraint is applied on the parameters governing kMT dynamics within the wild-type nucleus. The simplest model one can imagine for the mutant is that the non-essential KT proteins possibly affect at least one of the dynamic instability parameters, e.g., perhaps the catastrophe frequency is slightly lowered or equivalently the rescue frequency is slightly raised. Assuming constant values for parameters of kMT dynamics during anaphase and G1 (presented in Table S3), the distance between KT and SPB is measured and then the process is repeated for various initial configurations. Probability distributions of the distance are plotted in Figure S6B suggest that the position of the KT frequently goes past well beyond the diameter of the yeast nucleus (~2 μm).

**Text S5. Competing polar ejection force and kMT tension predicts mean KT position but does not qualify the coefficient of viscous drag**

Failure of the previous model leads us to attempt another model based on the “polar ejection force” [[22](#_ENREF_22),[25](#_ENREF_25)]. This force is primarily generated by the MTs, growing from the SPB and bumping into the chromosome arm at random instants. A MT, polymerizing in contact with the chromosome arm, transmit tiny pushing force to move the chromosome away from the SPB. This force is enhanced by chromosome bound chromokinesin motors that walk toward the plus end of the MT. The “polar ejection force” is counter balanced by the poleward tension between KT and SPB via the kMT adhesive/attachment complex. A schematic representation of this model is shown in Figure S7A.

Trajectory of MTs can be simulated using a Monte Carlo based algorithm in which MTs grow within a predefined solid angle subtended by the chromosomes at the SPB. Biased MT dynamics is not an essential criterion for this model; however, keeping in mind that the number of MTs involved in generating polar ejection force is small (~4), such an assumption will allow us to account for the maximum polar ejection force. Whenever, a growing MT hits the chromosome arm, it continues to push the chromosome away from SPB with a force ~3-4 pN [[26](#_ENREF_26),[27](#_ENREF_27)] before undergoing catastrophe. The magnitude and persistence of this force is enhanced by chromokinesin motors that walk toward the plus end of the MT while maintaining attachment with the chromosome arm. Upon catastrophe, MT depolymerizes and as soon as it shrinks completely, a new MT is nucleated in a random direction within the previously defined solid angle. Once a growing MT captures the KT, a poleward tension is established that helps the chromosome advance toward the SPB.

Polymerization and depolymerization of kMTs generate mechanical forces. These forces are transmitted to the KT by the linking protein complexes causing movement of KT. In the budding yeast, part of these linking protein complexes is believed to be a ring formed by oligomers of the Dam1 complex [[28](#_ENREF_28),[29](#_ENREF_29)]. If *F*_MT_ be the pushing force on the chromosome arm and *F*_KT_ represents the poleward tension during anaphase, the residue of the two opposing forces generates chromosomal motion of constant velocity given by *v*_KT_ = (*F*_MT_ - *F*_KT_)*/ς,* where ς is the viscous drag coefficient of the medium. *In vitro* experiments using reconstituted yeast KTs have shown that externally applied tension (mimicking metaphase) stabilizes kMT-KT attachment and increases its life-time as the tension is increased from 0.5 to 5 pN [[28](#_ENREF_28)], rupturing at 9 pN. Nevertheless, the maximum force that KTs experience in metaphase are several orders of magnitude larger than anaphase forces [[27](#_ENREF_27),[30](#_ENREF_30)]. For this simulation, we used 0.1 pN as the anaphase force on the KT per kMT (*F*_KT_) as calculated by Nicklas [[30](#_ENREF_30)]. After extensive literature search, we could not find the coefficient ς of the viscous drag for yeast nucleus and therefore used 5 pN-s/μm data, obtained from *Drosophila* chromosome [[31](#_ENREF_31)]. Substituting *F*_KT_ ~0.1 pN [[28](#_ENREF_28),[30](#_ENREF_30)] in the above equation, and using similar drag coefficients (ς ≤ 5 pN-s/μm), we calculated the distance of the chromosome from the SPB. Data plotted in Figure S7B features large amplitude fluctuations in the KT position. In order to minimize this fluctuation, we manipulated the simulation with viscous drag coefficients ς ~100 pN-s/μm, which is unrealistically large. Since the number of MTs involved in this process is small (~4), the probability that an MT tip impinge upon the chromosome arm per unit time is also very small. As mentioned earlier, the likelihood of this interaction can be increased by restricting the MTs within a solid angle directed toward the chromosomes. Simulating this model repeatedly by adjusting parameters over several orders of magnitude did not produce results that can explain the experimental data satisfactorily. Moreover, the presence of chromokinesin in yeast is largely debated. Thus, ‘polar ejection force’ model does not seem to be appropriate for the current purpose.

**Text S6. Physical interactions between SPB and KT components: possible significance**

An exhaustive survey of the *Saccharomyces* genome database (SGD) shows that a number of SPB central and inner plaque components interact physically with central or outer KT proteins (Table S2). Neither a physical interaction of an inner KT protein with an SPB component, nor that of an SPB outer plaque component with a KT structural protein was found. This indicates that the physical associations of KT proteins with SPB components are not entirely co-incidental, but may have biological significance. KTs are found clustered near SPBs almost throughout the cell cycle, a phenomenon that requires both functional microtubules (MTs) and KTs [[32](#_ENREF_32)]. KT clustering is suggested to help in accurate segregation of chromosomes by maintaining close proximity of SPB with segregating chromosomes [[19](#_ENREF_19),[33](#_ENREF_33),[34](#_ENREF_34),[35](#_ENREF_35),[36](#_ENREF_36)]. Since MT depolymerization by nocodazole does not completely abolish KT clustering, it has been suggested that residual clustering of KTs could be due to their physical associations with SPBs [[37](#_ENREF_37)]. It is difficult to envisage protein-protein interactions between SPB and KT components when the latter is MT-bound. Even two to three dimer lengths of the intervening MT (16-24 nm) would preclude direct contact between SPB and the KT. Nevertheless, it is probable that if the KT is detached from the MT, it could drift to SPB and get captured by it, even transiently, due to protein-protein interactions. The average distance between KTs and SPBs was found to be increased in the *chl4 ctf19* double mutant (Figure S3), suggesting that Chl4 and Ctf19 proteins help to maintain the distance at which KTs cluster around SPB. However, when cells were subjected to nocodazole to completely disrupt MTs in S-phase, the KT failed to localize at SPB even after 2 hours (Figure 2). Therefore, physical associations of Bbp1p with Chl4 and Ctf19 proteins did not promote KT clustering in any significant way. Another possible function of interactions between KT and SPB components could be to recruit KT proteins to the nucleus, in the vicinity of the spindle pole body. A higher concentration of KT proteins near the SPB could help in the speedy assembly of the central and outer layers of the KT complex on the newly replicated centromere DNA. Further experiments involving isolation of point mutations in *CHL4* and *CTF19* which disrupt the physical interactions of Chl4 and Ctf19 proteins with Bbp1p while retaining KT-MT interactions, and a study of their phenotypes in chromosome segregation, would throw more light on the biological significance of physical associations between KT and SPB proteins.

#
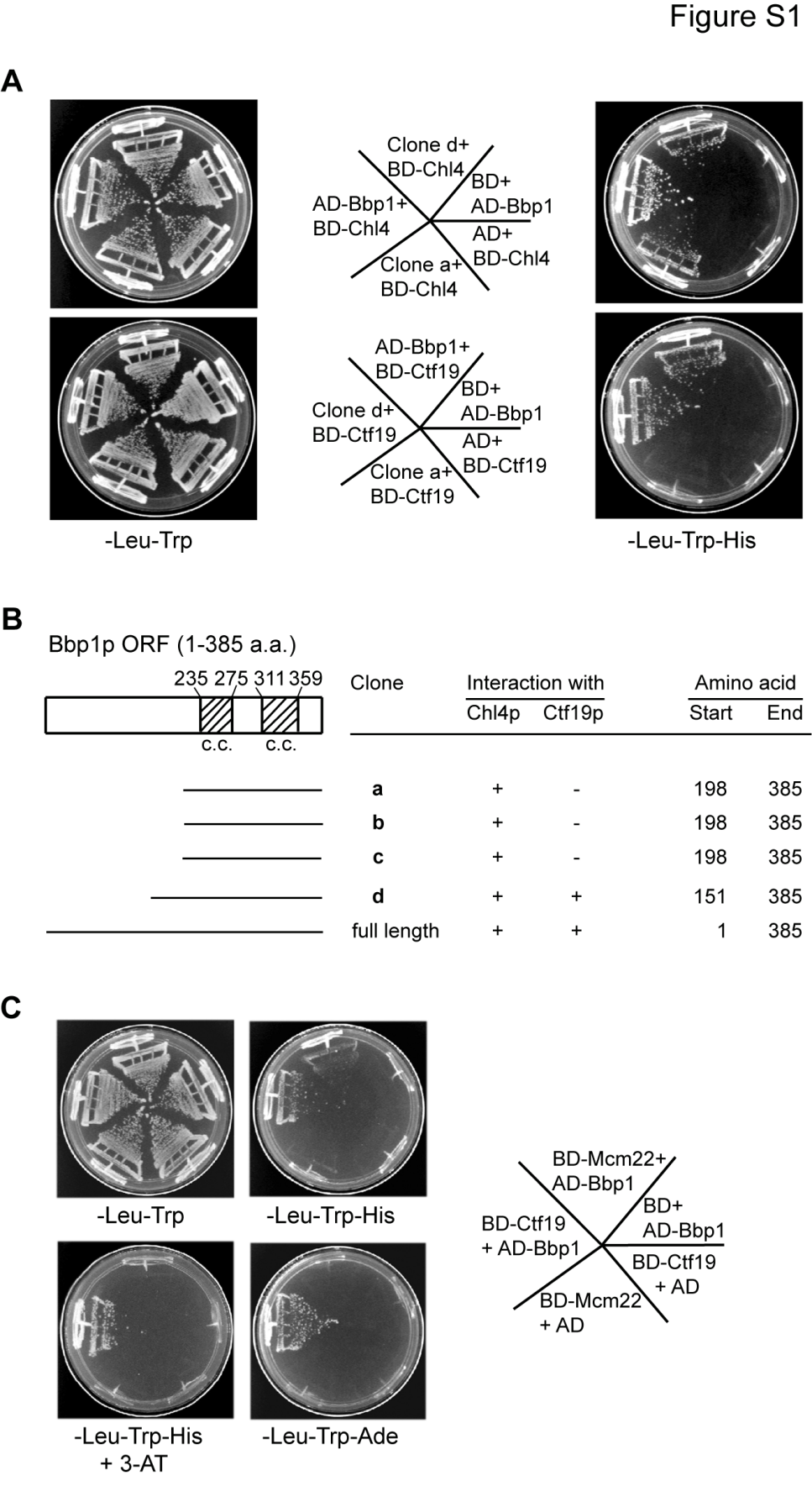


**Figure S1.** **Physical interaction of Chl4p and Ctf19p with Bbp1p.** (A) Physical interactions of Bbp1p with Chl4 and Ctf19 proteins. PJ69-4A was transformed with two-hybrid plasmids pGAD424 and pGBT9, the former expressing partial (clones **a** to **d)** or full length Bbp1p ORF fused to the *GAL4* activation domain, and the latter expressing Chl4p or Ctf19p fused to the *GAL4* binding domain. The transformants were selected on SC plates lacking leucine and tryptophan (-Leu-Trp). Freshly growing cells from these plates were streaked for growth at 30^ο^C on -Leu-Trp and SC plates lacking leucine, tryptophan and histidine (-Leu-Trp-His). Transformants growing on -Leu-Trp-His plates showed two-hybrid interactions between the fused proteins as described under methods. (B) Schematic diagram depicting different fragments of Bbp1p which interacted with Chl4 and Ctf19 proteins in two-hybrid screening. All the fragments contained two coiled-coil (c.c) domains at amino acids 235-275 and 311-359. +, two-hybrid interaction; -, no two-hybrid interaction. (C) Physical interaction of Mcm22p with Bbp1p. The PJ69-4A strain was transformed with two-hybrid plasmids expressing AD-Bbp1p and BD- Mcm22p. Other details were as described above.

**
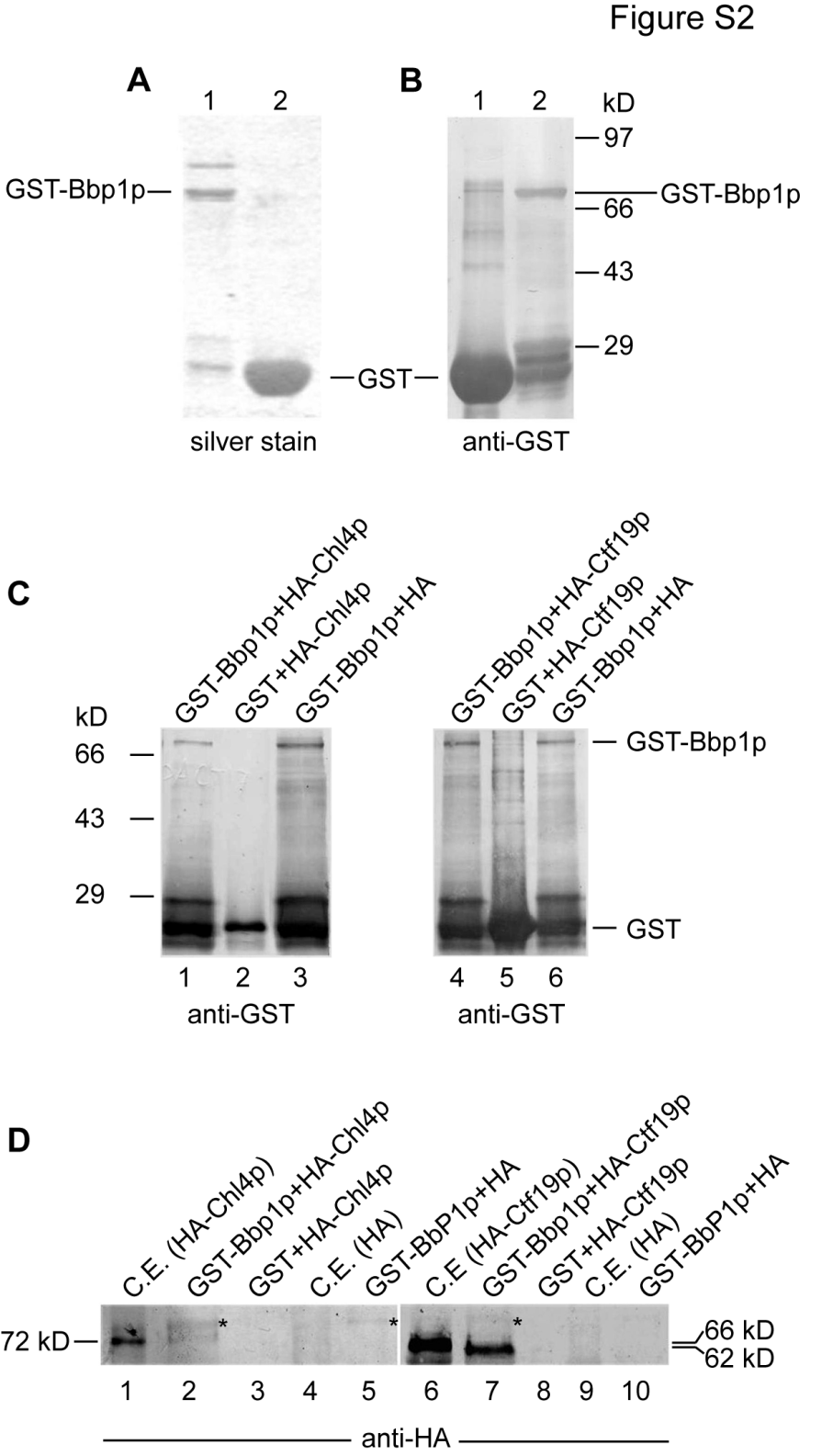
**

**Figure S2. Physical association of Chl4p and Ctf19p with Bbp1p *in vitro*.** (A) Purification of Bbp1p expressed in *E. coli.* Cell extracts were isolated from *E. coli* strain BL21 (DE3) carrying pGEX-5X-2 (vector) and pGEX-5X-2-*BBP1* and purified using glutathione Sepharose beads. Bound proteins were eluted with SDS-sample buffer and fractioned on a 10% polyacrylamide gel and silver stained. Lanes 1 and 2 are eluted GST-Bbp1p and GST fusion proteins, respectively. GST-Bbp1p fusion should migrate at 72 kD. (B) Immunoblot of Bbp1p using anti-GST antibody. The eluted proteins from Figure 2A were loaded in 10% polyacrylamide gel and immunoblotted using anti-GST antibody. Lanes 1 and 2 are purified GST and GST-Bbp1p fusion proteins, respectively. Protein size markers (kD) are also shown on the side. (C and D) *In vitro* association of Chl4p and Ctf19p with Bbp1p. AP22 (wild-type) strain was transformed separately with pACT2 expressing *HA*, *HA-CHL4* or *HA-CTF19* and GST pull-down studies were carried out as detailed in Materials and methods, by mixing cell extracts obtained from each transformant with equal amounts of purified bead-bound GST or GST-Bbp1p. Proteins eluted from glutathione Sepharose beads were loaded on 10% polyacrylamide gels for Western blotting, as described under methods. (C) Anti-GST immunoblot, for detecting GST-Bbp1p and GST proteins in GST pull-down fractions. (D) Anti-HA immunoblot, for detecting HA, HA-Chl4p and HA-Ctf19p in GST pull-down fractions. Lanes 1 and 6 show protein bands corresponding to HA-Chl4p (72 kD) and HA-Ctf19p (62 kD), respectively, in the cell extracts of the corresponding transformants. Asterix indicates a non-specific band that tends to appear in lanes carrying GST-Bbp1p when probed with anti-HA.

**
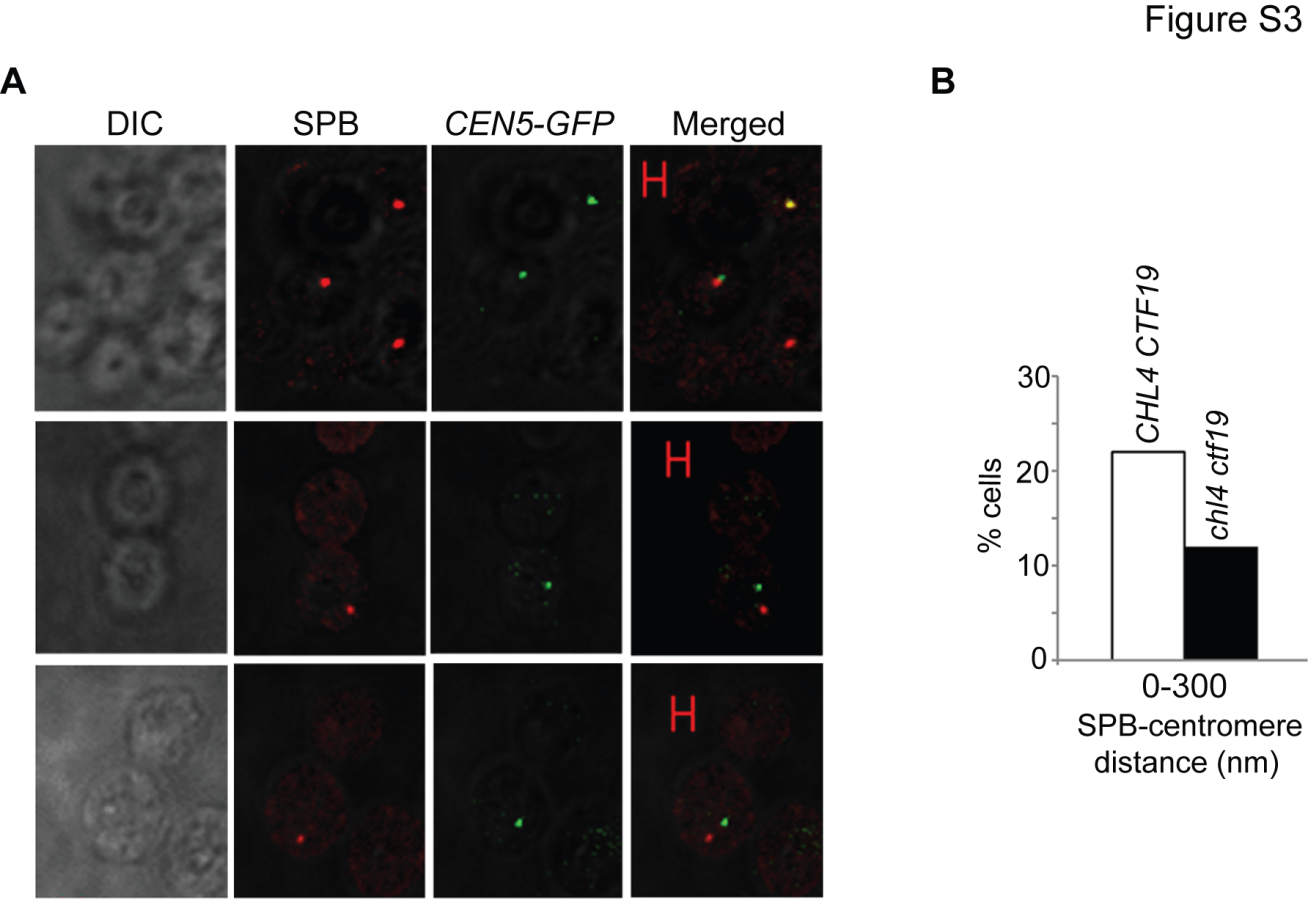
**

**Figure S3. Wild-type centromeres lie closer to SPBs than mutant centromeres upon MT disruption by nocodazole.** Exponentially growing cells of US3329 (wild-type) and US3329∆ctf19Dchl4 (*chl4 ctf19*) in YEPD at 30^ο^C were arrested at G2/M using nocodazole (15 μg/ml) for 120 minutes. Cells were fixed with formaldehyde and anti-α-tubulin was used to stain SPB, which appeared as a round spot in about 20-30% of the cells. Images were captured in 0.5 μm z-sections and analyzed by confocal microscopy. (A) Upper panel: A section of z-stack is shown containing three large budded cells with SPBs visible as red dots, but with no visible spindles. In the same section, one cell also shows a *CEN5-*GFP which is co-localized with SPB (yellow spot in merged figure), another cell shows non-overlapping SPB and *CEN5*-GFP dots and the third cell has its *CEN5*-GFP dot in the next section of z-stack (not shown). Middle and lower panels: Cells are shown with well-separated *CEN5*-GFP and SPB dots. The horizontal scale bar represents 1 μm. (B) The bar diagram represents percentages of cells in which the 3D distance between *CEN5* and SPB lies within 300 nm.

**
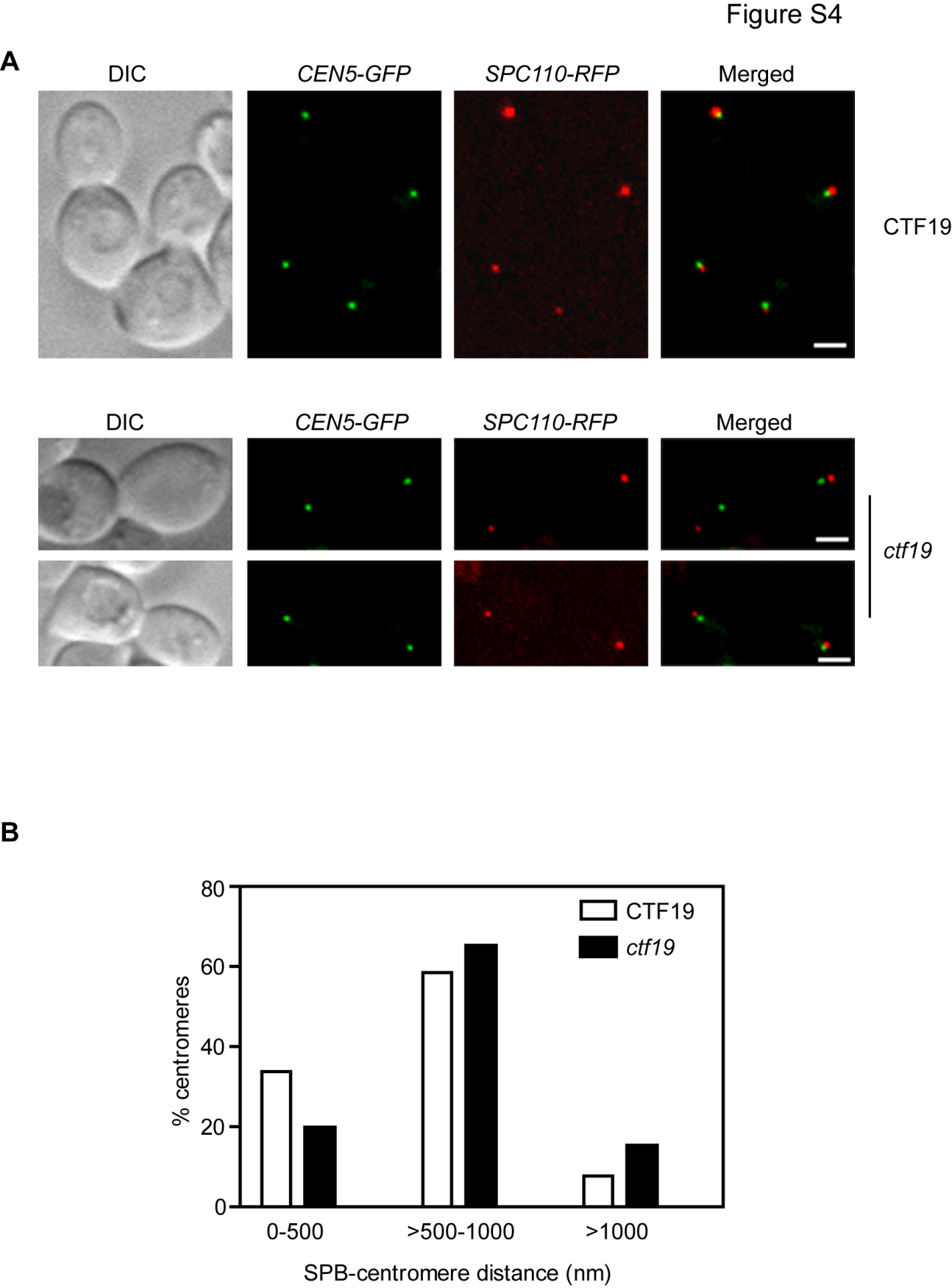
**

**Figure S4. *ctf19* anaphase cells without nocodazole treatment show increased SPB-KT distance over corresponding wild-type cells.** Exponentially growing wild-type *CTF19* (PS1) and mutant *ctf19* (PS1Δctf19) cells were arrested in G1 by α-factor and, after 145 minutes of release from the arrest, they were harvested and treated for microscopy as described earlier (Materials and methods). The synchronization with α-factor increased the proportion of anaphase B cells in the culture. SPB-KT distances were measured as described in Materials and methods for this figure. (A) Cells showing the separation of SPBs (red) from their centromeres (green). Upper panel, wild-type cells; lower panel, mutant *ctf19* cells. Scale bars, 2 μm. (B) The histogram shows the distribution of centromeres based on their distances from their SPBs. The average distances for the wild-type and the mutant strains were 620±241 and 710±349 nm, respectively. 65 centromeres were analysed from the wild-type and 66 from the mutant strain.

Note: The fraction of wild-type kinetochores lying within 500 nm of their SPBs was about 1.7 times the corresponding fraction of mutant kinetochores, which is similar to that obtained for nocodazole-treated cells (Figure 1B). However, the absolute values of SPB-KT distances for both the strains are higher than those obtained for nocodazole-treated cells (Figure 1). We believe that this could be due to one or both of the following reasons. One, the Spc110p-RFP tag used here for locating the SPB is at the C-terminus of the protein which lies at the central plaque of the SPB, about 100 nm away from the inner plaque [[38](#_ENREF_38)]. In the earlier experiment (Figure 1) SPB was identified by α-tubulin antibody which stains the inner plaque within the nucleus, thereby increasing the SPB-KT distance by about 100 nm in the present experiment over the earlier one. The second reason could be the use of a different microscope and its software for measuring distances in the present experiment, since RFP fluorescence could not be detected satisfactorily by the confocal microscope used in the earlier experiment.

**
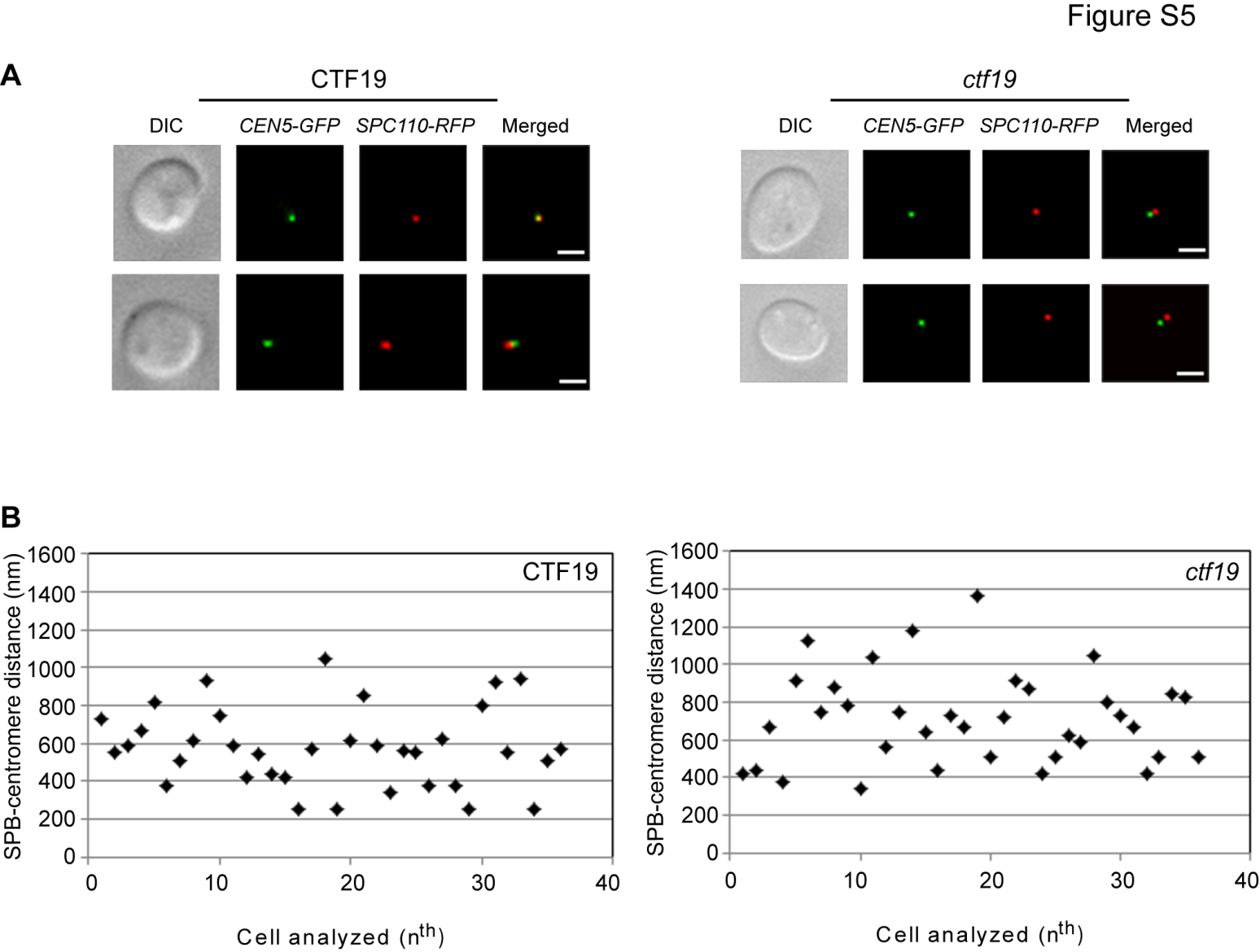
**

**Figure S5. G1 cells of asynchronous *ctf19* culture show increased SPB-KT distance compared to corresponding wild-type cells.** Exponentially growing cells of *CTF19* (PS1) and *ctf19* (PS1Δctf19) strains were treated for fluorescence microscopy (Materials and methods). G1 cells were identified as being round and unbudded. SPB-KT distances were measured as described in Materials and methods for this figure. (A) G1 cells illustrating separation between SPB (red) and centromere (green) in wild-type (upper panel) and mutant (lower panel) cells. Scale bars, 2 μm. (B) Scatter plots showing the distance of each centromere from its SPB. 36 cells were analysed in each case. The average distances were 570±211 nm (wild-type) and 709±244 nm (mutant).

**
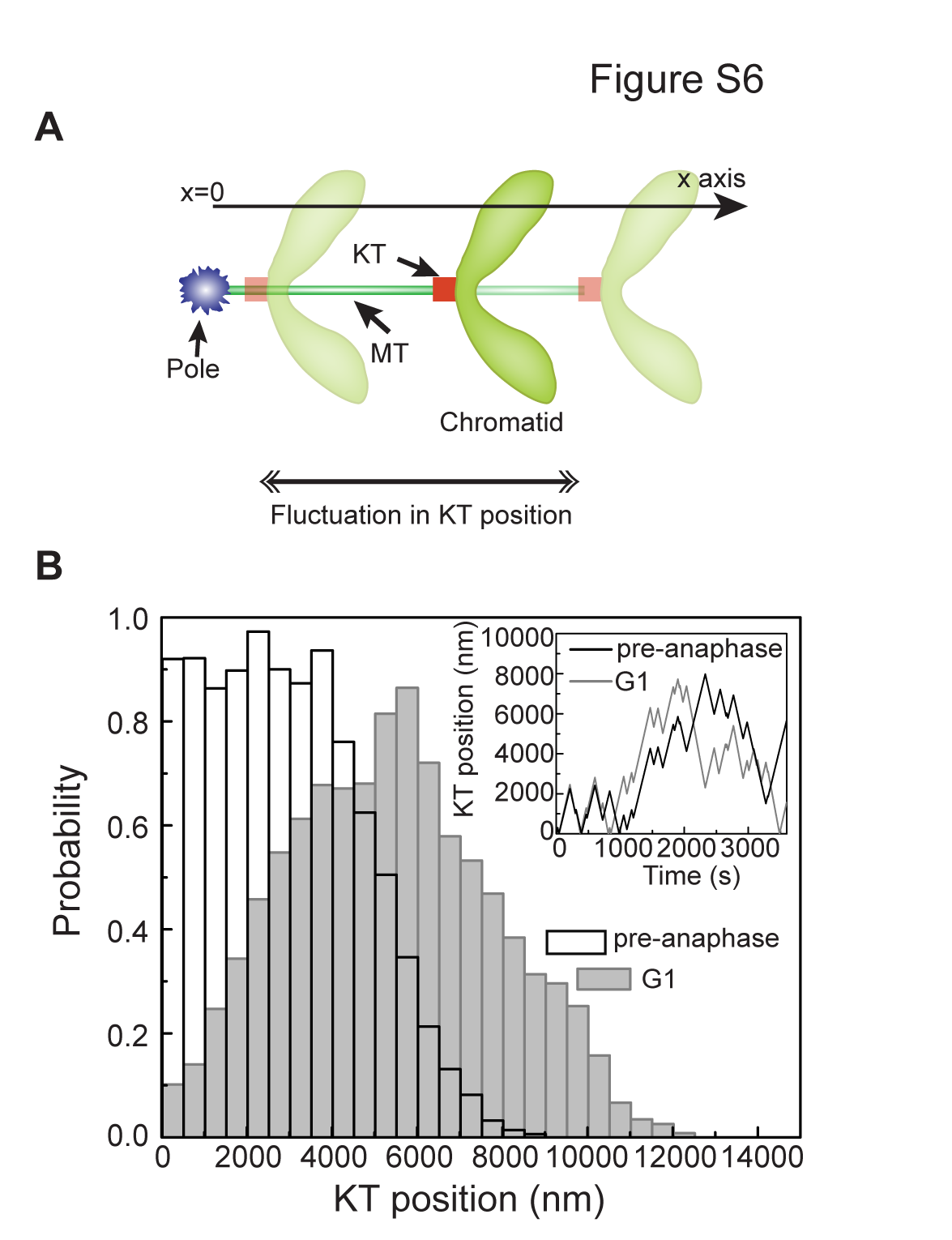
**

Figure S6. Unconstrained MT dynamics derived KT positioning. (A) Schematic of the model showing a KT attached with a single MT moves toward and away from the SPB (pole) coherently with the growth and shortening of the kMT. Three different positions of the KT are shown. (B) Probability distribution of the KT position measured from the SPB for anaphase and G1. Inset shows the time dependent trajectory of a KT.

**
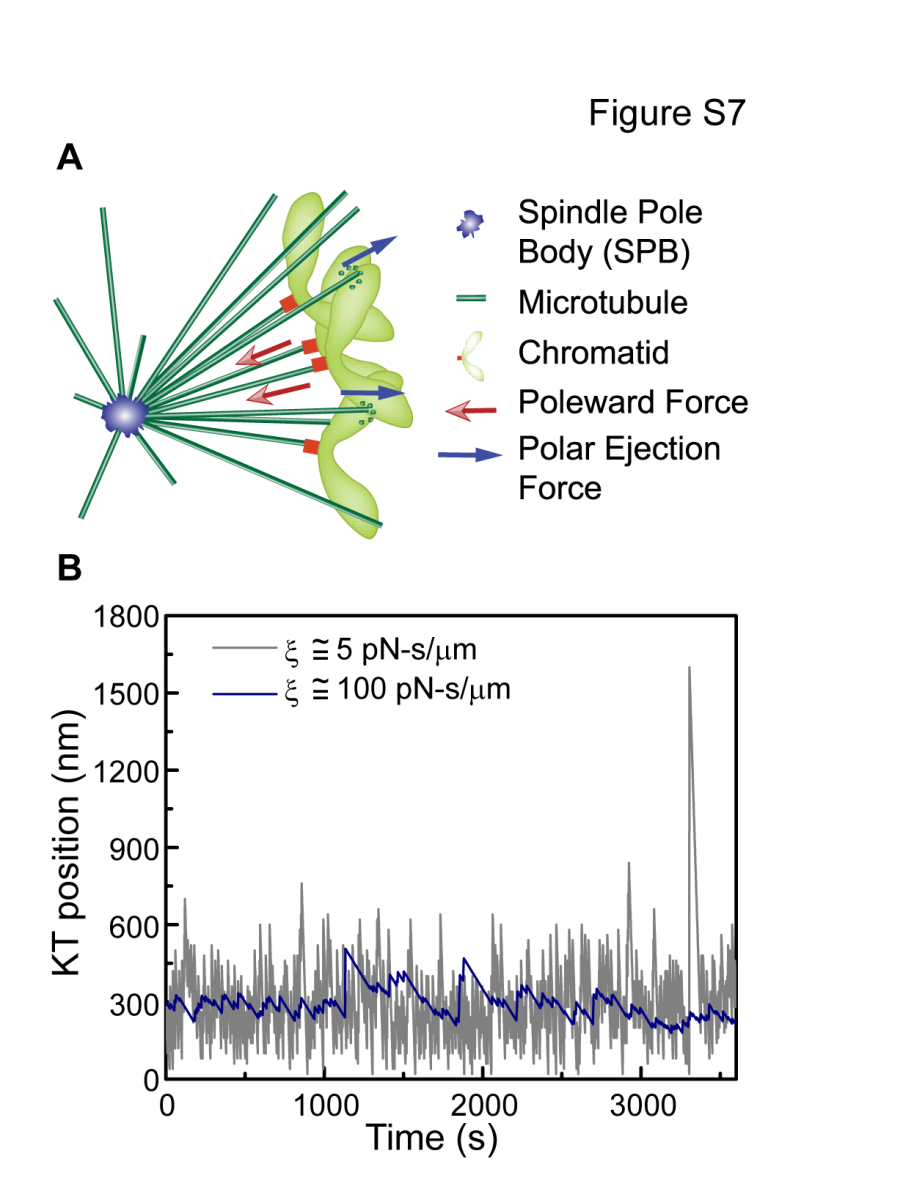
**

Figure S7. Competing ‘polar ejection force’ and kMT tension requires high viscosity to predict mean KT position. (A) Working model: Blue sphere represents the SPB and the green rods emerging from the SPB depict MTs. MTs generate ‘polar ejection force’ as they impinge upon the chromosome arm (light green structure). This force is enhanced by plus end directed chromokinesin motors acting at the overlap between MT and chromosome arm. Red arrows show the direction of the poleward tension between KT and SPB. (B) Position of the KT, measured from the SPB, is plotted as a function of time for different values of the viscous drag coefficient (ς). A large spatial fluctuation (~ ±300 nm) about the mean KT position is observed for ς ~5 pN-µm/s. Such fluctuations can be reduced only if a very large value of the ς ~100 pN-µm/s is used in the simulation.

**
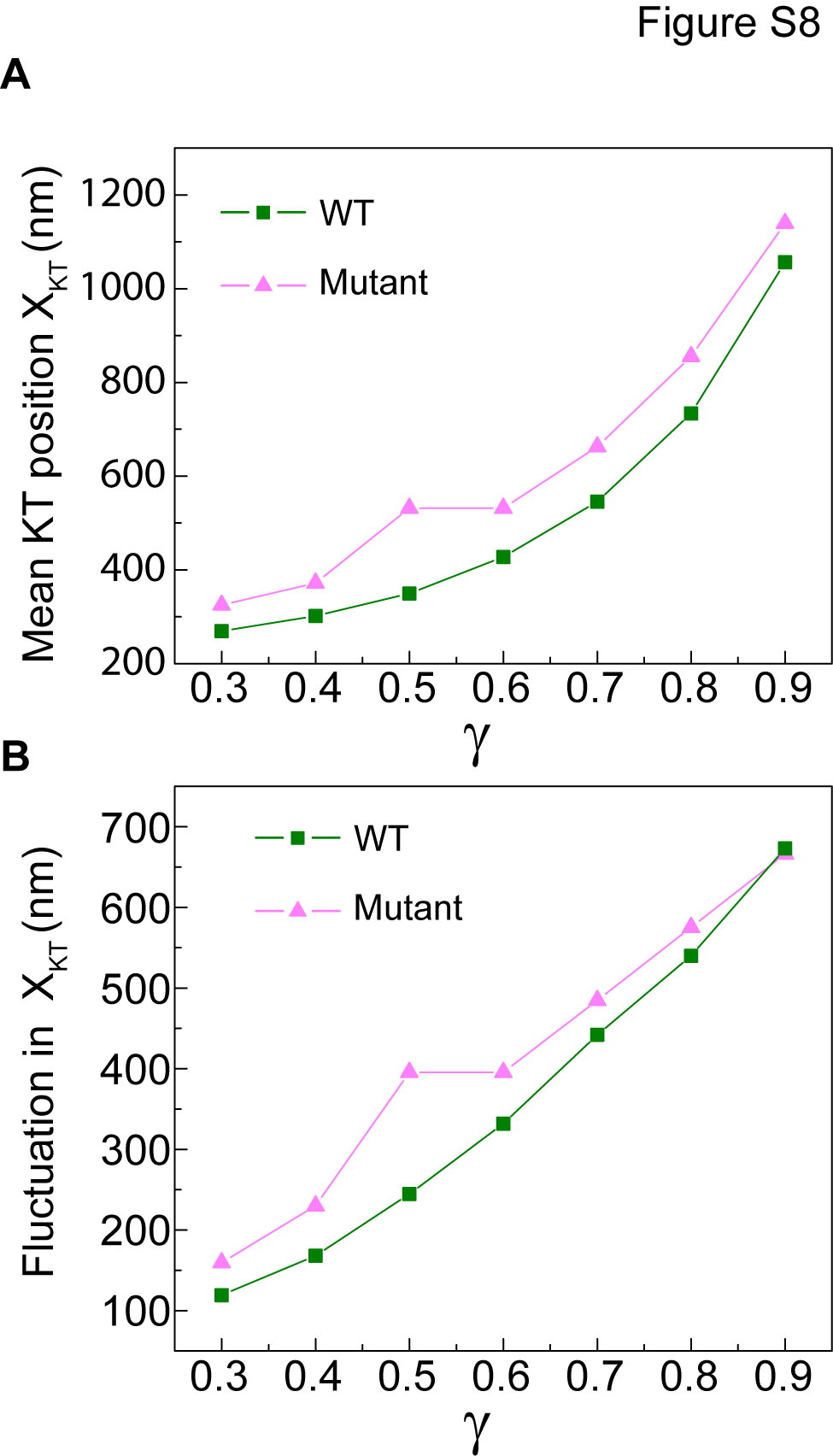
**

Figure S8. Mean KT position; fluctuation increases with efficiency (γ) of the KT being pushed by kMT. (A) Mean position of the KT (X_KT_) measured from SPB is plotted against γ increases for both WT and mutant cells. For small γ, mutant KT position is further from the SPB than the wild-type. Increasing γ effectively stiffens the linker configuration amplifying the catastrophe frequency of the MT. For γ close to unity the linker proteins do not distinguish between a WT and mutant and therefore the difference between the mean KT positions also vanishes. (B) Fluctuation in the mean KT position [measured from the full width at half maxima (FWHM) of the Gaussian fits] increases with γ.

**
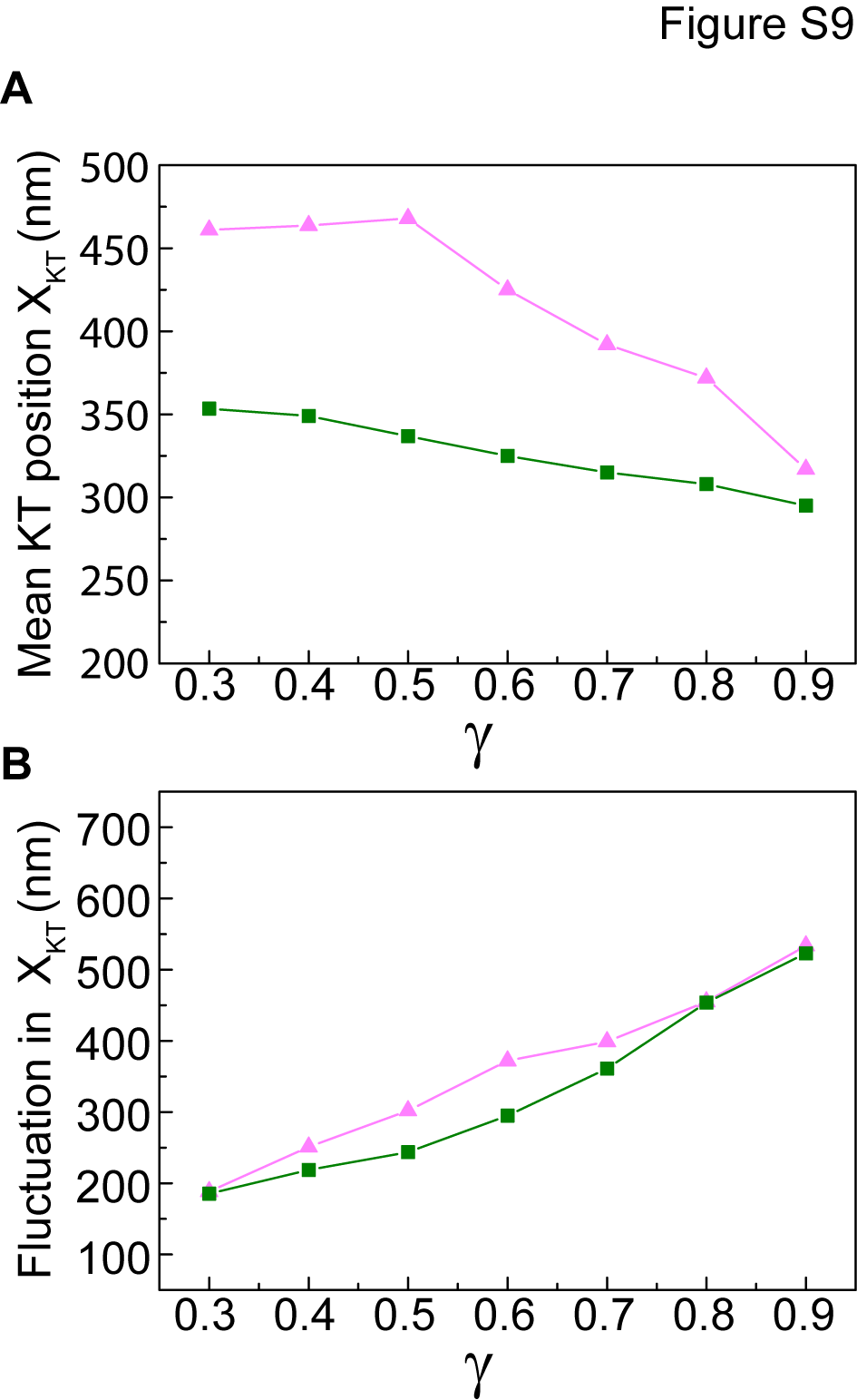
**

Figure S9. Mean KT position; fluctuation increases with efficiency (γ) of the KT being pulled by kMT. (A) Mean position of the KT (X_KT_) measured from SPB is plotted against γ decreases for both WT and mutant cells. Difference in the KT position between wild type and mutant is reduced for large values of γ. In the limit γ close to unity the linker proteins do not distinguish between a WT and mutant and therefore the difference between the mean KT positions also vanishes. (B) Fluctuation in the mean KT position [measured from the full width at half maxima (FWHM) of the Gaussian fits] increases with γ.

**
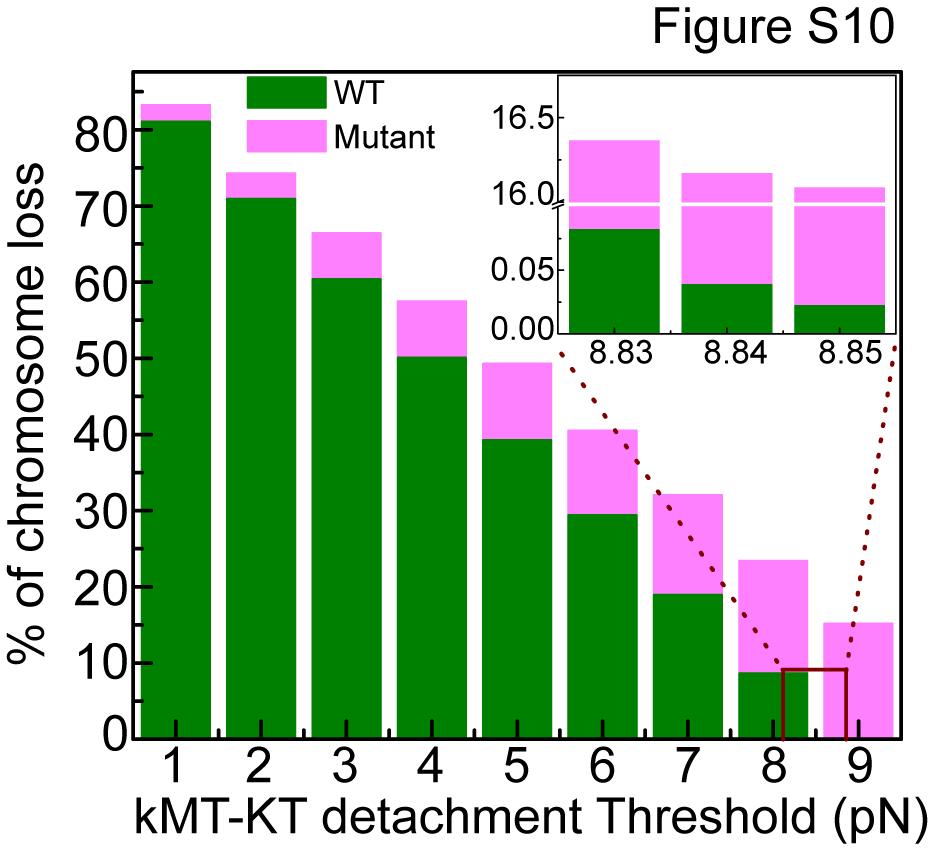
**

Figure S10. Chromosome loss statistics. Percentage of chromosome detached from the kMT detachment force. KT detaches from kMT once the tension between them exceeds a threshold. Data shown here is obtained from a simulation over 100000 samples. We find that in the wild-type (WT) cell, ~0.03% chromosome is lost for a threshold tension ~8.85 pN. For the same magnitude of threshold, chromosome loss for the mutant cell is ~16%. Our model prediction for the WT cell agrees with the experimental results [[19](#_ENREF_19)].

**Table S1. Strains used in this study.**

| **Strain** | **Genotype** | **Deletion/disruption strategy [reference]** |
| --- | --- | --- |
| AP22 | *MATα* *leu2-3,112 his3-11,15 ura3-52 trp1* | [[39](#_ENREF_39)] |
| AP22*∆*iml3 | *MATα* *leu2-3,112 his3-11,15 ura3-52 trp1 iml3-Δ1::URA3* | By deleting *IML3* in AP22 [[5](#_ENREF_5)] |
| PJ69-4A | *MAT***a** *trp1-901 leu2-3,112 ura3-52 his3-200 gal4∆ gal80∆ LYS2::GAL1-HIS3 GAL2-ADE2 met2::GAL7-lacZ* | [[1](#_ENREF_1)] |
| PJ69-4A*∆*16 | PJ69-4A *mcm16-Δ1::URA3* | By deleting *MCM16* in PJ69-4A [[9](#_ENREF_9)] |
| PJ69-4A∆chl4 | PJ69-4A *chl4-Δ1::URA3* | By deleting *CHL4* in PJ69-4A [[40](#_ENREF_40)] |
| PJ69-4A∆ctf19 | PJ69-4A *ctf19-Δ1::URA3* | This study, by deleting *CTF19* in PJ69-4A |
| PJ69-4A∆iml3 | PJ69-4A *iml3-Δ1::URA3* | By deleting *IML3* in PJ69-4A [[5](#_ENREF_5)] |
| PJ69-4A∆chl4∆iml3 | PJ69-4A *chl4-Δ1::URA3 iml3-Δ1::KANMX6* | This study, by deleting *IML3* in PJ69-4A∆chl4 |
| PJ69-4AD21 | PJ69-4A *mcm21::URA3* | By disrupting *MCM21* in PJ69-4A [[6](#_ENREF_6)] |
| PJ69-4AD22 | PJ69-4A *mcm22::HIS3* | By disrupting *MCM22* in PJ69-4A [[6](#_ENREF_6)] |
| US3329 | *MAT***a** *leu2::LEU2::tetR-GFP tetOX224::HIS3 ura3 trp1 leu2 his3 ade2* | [[41](#_ENREF_41)] |
| US3329∆chl4 | *MAT***a** *leu2::LEU2::tetR-GFP tetOX224::HIS3 ura3 trp1 leu2 his3 ade2 chl4-Δ1::URA3* | This study, by deleting *CHL4* in US3329 |
| US3329∆ctf19 | *MAT***a** *leu2::LEU2::tetR-GFP tetOX224::HIS3 ura3 trp1 leu2 his3 ade2 ctf19-Δ1::URA3* | This study, by deleting *CTF19* in US3329 |
| US3329∆ctf19Dchl4 | *MAT***a** *leu2::LEU2::tetR-GFP tetOX224::HIS3 ura3 trp1 leu2 his3 ade2 ctf19-Δ1::URA3 chl4::TRP1* | This study, by disrupting *CHL4* in US3329∆ctf19 |
| CCY 1782-2B | *MAT***a** *his3 leu2-3, 112 ura3 trp1-1*  *SPC110-RFP::kanMX* | Clarence Chan |
| US3759 | *MAT*α *trp1bar1∆::URA3, tetR-GFP::LEU2 tetO::CEN5::HIS3*  *SPC29-CFP::KAN* | Uttam Surana |
| PS1 | *MAT***a** *ura3 trp1-1 tetR-GFP::LEU2 tetO::CEN5::HIS3 SPC110-RFP::kanMX* | This study, by crossing US3759 with CCY1782-2B |
| PS1Δctf19 | *MAT***a** *ura3 trp1-1 tetR-GFP::LEU2 tetO::CEN5::HIS3 SPC110-RFP::kanMX ctf19::URA3* | This study, by deleting *CTF19* in PS1 |
| SS3 | AP22 *BBP1-13Myc- His3MX6* | This study |

**Table S2.** **Physical interactions between KT and SPB proteins.**

| SPB | KT | | Assay | Reference |
| --- | --- | --- | --- | --- |
| Protein, Location | Protein | Location, complex |  |  |
| Bbp1, periphery  of the central plaque, some in the inner plaque | Chl4  Ctf19  Nkp2 | All three localized at central KT, Ctf19 complex | Two-hybrid,  GST pull-down  Two-hybrid | This study  [[20](#_ENREF_20)] |
| Mps2, central plaque, nuclear envelope | Spc24  Nnf1 | Central KT, Ndc80 complex  Central KT, MIND complex | Two-hybrid, Co-IP  Two-hybrid | [[20](#_ENREF_20),[37](#_ENREF_37)]  [[20](#_ENREF_20),[42](#_ENREF_42)] |
| Nbp1, central plaque | Ctf19 | Central KT,  Ctf19 complex | Two-hybrid | [[20](#_ENREF_20)] |
| Spc29, inner plaque | Nuf2 | Central KT, Ndc80 complex | Two-hybrid | [[42](#_ENREF_42)] |
| Spc42, central plaque | Nuf2  Spc19 | Central KT, Ndc80 complex  Outer KT, DASH complex | Two-hybrid  Two-hybrid | [[42](#_ENREF_42)]  [[20](#_ENREF_20)] |
| Spc110, inner plaque | Nuf2  Ndc80  Spc24  Spc25  Spc105  Spc19  Spc34 | Central KT  Outer KT, DASH complex | Co-purification  Co-purification | [[15](#_ENREF_15)]  [[15](#_ENREF_15)] |
| Mps3, half bridge protein | Ctf19 | Central KT, Ctf19 complex | Two-hybrid | [[20](#_ENREF_20)] |

Information on the localizations of SPB and KT proteins and the interactions between them were obtained from “*Saccharomyces* Genome Database” http://www. yeastgenome.org

**Table S3**

**Model parameters:**

| Abbreviations | Meaning | Values used | Reference |
| --- | --- | --- | --- |
| *v*_0_ | Unconstrained growth velocity | 0.79 µm/min ^a^  0.85 µm/min ^b^ | [[43](#_ENREF_43)] |
| *v*_s_ | Depolymerizing velocity | 0.86 µm/min ^a^  1.04 µm/min ^b^ | [[43](#_ENREF_43)] |
| __ | Catastrophe frequency | 0.0109/s ^a^  0.0104/s ^b^ | [[43](#_ENREF_43)] |
| __ | Rescue frequency | 0.0089/s ^a^  0.0130/s ^b^ | [[43](#_ENREF_43)] |
| *l*_KT_ | length of the KT | 0.1 µm | [[44](#_ENREF_44)] |

^a^ Values used for anaphase

^b^ Values used for G1

**Supplemental References**

1. James P, Halladay J, Craig EA (1996) Genomic Libraries and a Host Strain Designed for Highly Efficient Two-Hybrid Selection in Yeast. Genetics 144: 1425-1436.

2. Bradford MM (1976) A rapid and sensitive method for the quantitation of microgram quantities of protein utilizing the principle of protein-dye binding. Anal Biochem 72: 248-254.

3. Maniatis T FEF, Sambrook J (1982) Molecular Cloning: A Laboratory Manual, Cold Spring Harbor Laboratory Press, New York.

4. Laha S, Das SP, Hajra S, Sau S, Sinha P (2006) The budding yeast protein Chl1p is required to preserve genome integrity upon DNA damage in S-phase. Nucleic Acids Res 34: 5880-5891.

5. Ghosh SK, Poddar A, Hajra S, Sanyal K, Sinha P (2001) The IML3/MCM19 gene of Saccharomyces cerevisiae is required for a kinetochore-related process during chromosome segregation. Mol Genet Genomics 265: 249-257.

6. Poddar A, Roy N, Sinha P (1999) MCM21 and MCM22, two novel genes of the yeast Saccharomyces cerevisiae are required for chromosome transmission. Mol Microbiol 31: 349-360.

7. Wach A, Brachat A, Pöhlmann R, Philippsen P (1994) New heterologous modules for classical or PCR-based gene disruptions in Saccharomyces cerevisiae. Yeast 10: 1793-1808.

8. Gietz RD, Akio S (1988) New yeast-Escherichia coli shuttle vectors constructed with in vitro mutagenized yeast genes lacking six-base pair restriction sites. Gene 74: 527-534.

9. Sanyal K, Ghosh SK, Sinha P (1998) The MCM16 gene of the yeast Saccharomyces cerevisiae is required for chromosome segregation. Mol Gen Genet 260: 242-250.

10. Bartel PL CC-T, Sternglanz R, Fields S (1993) Using the two-hybrid system to detect protein-protein inetractions. in: D.A. Harely (Ed.), Cellular interactions in development: a practical approach, Oxford University Press, Oxford.

11. Hajra S (2003) Kinetochore structure of the budding yeast *Saccharomyces cerevisiae*: A study using genetic and protein-protein interactions, Ph. D thesis, Jadavpur University, Kolkata, India.

12. Li L, Elledge SJ, Peterson CA, Bales ES, Legerski RJ (1994) Specific association between the human DNA repair proteins XPA and ERCC1. Proc Natl Acad Sci U S A 91: 5012-5016.

13. Longtine MS, McKenzie Iii A, Demarini DJ, Shah NG, Wach A, et al. (1998) Additional modules for versatile and economical PCR-based gene deletion and modification in Saccharomyces cerevisiae. Yeast 14: 953-961.

14. Stover CK, de la Cruz VF, Fuerst TR, Burlein JE, Benson LA, et al. (1991) New use of BCG for recombinant vaccines. Nature 351: 456-460.

15. Wigge PA, Jensen ON, Holmes S, Souès S, Mann M, et al. (1998) Analysis of the Saccharomyces Spindle Pole by Matrix-assisted Laser Desorption/Ionization (MALDI) Mass Spectrometry. J Cell Biol 141: 967-977.

16. Measday V, Hailey DW, Pot I, Givan SA, Hyland KM, et al. (2002) Ctf3p, the Mis6 budding yeast homolog, interacts with Mcm22p and Mcm16p at the yeast outer kinetochore. Genes Dev 16: 101-113.

17. Pot I, Measday V, Snydsman B, Cagney G, Fields S, et al. (2003) Chl4p and Iml3p Are Two New Members of the Budding Yeast Outer Kinetochore. Mol Biol Cell 14: 460-476.

18. Ortiz J, Stemmann O, Rank S, Lechner J (1999) A putative protein complex consisting of Ctf19, Mcm21, and Okp1 represents a missing link in the budding yeast kinetochore. Genes Dev 13: 1140-1155.

19. Hyland KM, Kingsbury J, Koshland D, Hieter P (1999) Ctf19p: A Novel Kinetochore Protein in Saccharomyces cerevisiae and a Potential Link between the Kinetochore and Mitotic Spindle. J Cell Biol 145: 15-28.

20. Wong J, Nakajima Y, Westermann S, Shang C, Kang J-s, et al. (2007) A Protein Interaction Map of the Mitotic Spindle. Mol Biol Cell 18: 3800-3809.

21. Schramm C, Elliott S, Shevchenko A, Shevchenko A, Schiebel E (2000) The Bbp1p-Mps2p complex connects the SPB to the nuclear envelope and is essential for SPB duplication. EMBO J 19: 421-433.

22. Rieder CL, Davison EA, Jensen LC, Cassimeris L, Salmon ED (1986) Oscillatory movements of monooriented chromosomes and their position relative to the spindle pole result from the ejection properties of the aster and half-spindle. J Cell Biol 103: 581-591.

23. Kirschner M, Mitchison T (1986) Beyond self-assembly: From microtubules to morphogenesis. Cell 45: 329-342.

24. Mitchison T, Kirschner M (1984) Dynamic instability of microtubule growth. Nature 312: 237-242.

25. Levesque AA, Compton DA (2001) The chromokinesin Kid is necessary for chromosome arm orientation and oscillation, but not congression, on mitotic spindles. J Cell Biol 154: 1135-1146.

26. Dogterom M, Yurke B (1997) Measurement of the Force-Velocity Relation for Growing Microtubules. Science 278: 856-860.

27. Inoué S, Salmon ED (1995) Force generation by microtubule assembly/disassembly in mitosis and related movements. Mol Biol Cell 6: 1619-1640.

28. Akiyoshi B, Sarangapani KK, Powers AF, Nelson CR, Reichow SL, et al. (2010) Tension directly stabilizes reconstituted kinetochore-microtubule attachments. Nature 468: 576-579.

29. Franck AD, Powers AF, Gestaut DR, Gonen T, Davis TN, et al. (2007) Tension applied through the Dam1 complex promotes microtubule elongation providing a direct mechanism for length control in mitosis. Nat Cell Biol 9: 832-837.

30. Nicklas RB (1988) The Forces that Move Chromosomes in Mitosis. Annu Rev Biophys Biophys Chem 17: 431-449.

31. Marshall WF, Marko JF, Agard DA, Sedat JW (2001) Chromosome elasticity and mitotic polar ejection force measured in living Drosophila embryos by four-dimensional microscopy-based motion analysis. Curr Biol 11: 569-578.

32. Jin QW, Fuchs J, Loidl J (2000) Centromere clustering is a major determinant of yeast interphase nuclear organization. J Cell Sci 113: 1903-1912.

33. Guacci V, Hogan E, Koshland D (1997) Centromere position in budding yeast: evidence for anaphase A. Mol Biol Cell 8: 957-972.

34. Janke C, Ortiz J, Lechner J, Shevchenko A, Shevchenko A, et al. (2001) The budding yeast proteins Spc24p and Spc25p interact with Ndc80p and Nuf2p at the kinetochore and are important for kinetochore clustering and checkpoint control. EMBO J 20: 777-791.

35. Jin Q-w, Trelles-Sticken E, Scherthan H, Loidl J (1998) Yeast Nuclei Display Prominent Centromere Clustering That Is Reduced in Nondividing Cells and in Meiotic Prophase. J Cell Biol 141: 21-29.

36. Hou H, Zhou Z, Wang Y, Wang J, Kallgren SP, et al. (2012) Csi1 links centromeres to the nuclear envelope for centromere clustering. J Cell Biol 199: 735-744.

37. Masson IL, Saveanu C, Chevalier A, Namane A, Gobin R, et al. (2002) Spc24 interacts with Mps2 and is required for chromosome segregation, but is not implicated in spindle pole body duplication. Mol Microbiol 43: 1431-1443.

38. Winey M, Bloom K (2012) Mitotic Spindle Form and Function. Genetics 190: 1197-1224.

39. Das SP, Sinha P (2005) The budding yeast protein Chl1p has a role in transcriptional silencing, rDNA recombination, and aging. Biochem Biophys Res Commun 337: 167-172.

40. Roy N, Poddar A, Lohia A, Sinha P (1997) The mcm17 mutation of yeast shows a size-dependent segregational defect of a mini-chromosome. Curr Genet 32: 182-189.

41. Ghosh SK, Sau S, Lahiri S, Lohia A, Sinha P (2004) The Iml3 protein of the budding yeast is required for the prevention of precocious sister chromatid separation in meiosis I and for sister chromatid disjunction in meiosis II. Curr Genet 46: 82-91.

42. Newman JRS, Wolf E, Kim PS (2000) A computationally directed screen identifying interacting coiled coils from Saccharomyces cerevisiae. Proc Natl Acad Sci USA 97: 13203-13208.

43. Wolyniak MJ, Blake-Hodek K, Kosco K, Hwang E, You L, et al. (2006) The Regulation of Microtubule Dynamics in Saccharomyces cerevisiae by Three Interacting Plus-End Tracking Proteins. Mol Biol Cell 17: 2789-2798.

44. Gonen S, Akiyoshi B, Iadanza MG, Shi D, Duggan N, et al. (2012) The structure of purified kinetochores reveals multiple microtubule-attachment sites. Nat Struct Mol Biol 19: 925-929.
